# Bacterial Diseases of Bananas and Enset: Current State of Knowledge and Integrated Approaches Toward Sustainable Management

**DOI:** 10.3389/fpls.2017.01290

**Published:** 2017-07-20

**Authors:** Guy Blomme, Miguel Dita, Kim Sarah Jacobsen, Luis Pérez Vicente, Agustin Molina, Walter Ocimati, Stephane Poussier, Philippe Prior

**Affiliations:** ^1^Bioversity International Addis Ababa, Ethiopia; ^2^Brazilian Agricultural Research Corporation – Embrapa Cassava and Fruits Cruz das Almas, Brazil; ^3^Royal Museum for Central Africa Tervuren, Belgium; ^4^Institute of Plant Health Research, Ministry of Agriculture Havana, Cuba; ^5^Bioversity International Los Baños, Philippines; ^6^Bioversity International Kampala, Uganda; ^7^UMR PVBMT, University of Reunion La Réunion, France; ^8^UMR PVBMT, CIRAD-INRA La Réunion, France

**Keywords:** bacterial disease, banana, ensete, Ralstonia-associated, Xanthomonas wilt, Erwinia-associated

## Abstract

Bacterial diseases of bananas and enset have not received, until recently, an equal amount of attention compared to other major threats to banana production such as the fungal diseases black leaf streak (*Mycosphaerella fijiensis*) and Fusarium wilt (*Fusarium oxysporum* f. sp. *cubense*). However, bacteria cause significant impacts on bananas globally and management practices are not always well known or adopted by farmers. Bacterial diseases in bananas and enset can be divided into three groups: (1) Ralstonia-associated diseases (Moko/Bugtok disease caused by *Ralstonia solanacearum* and banana blood disease caused by *R. syzygii* subsp. *celebesensis*); (2) Xanthomonas wilt of banana and enset, caused by *Xanthomonas campestris* pv. *musacearum* and (3) Erwinia-associated diseases (bacterial head rot or tip-over disease *Erwinia carotovora* ssp. *carotovora* and *E. chrysanthemi*), bacterial rhizome and pseudostem wet rot (*Dickeya paradisiaca* formerly *E. chrysanthemi* pv. *paradisiaca*). Other bacterial diseases of less widespread importance include: bacterial wilt of abaca, Javanese vascular wilt and bacterial fingertip rot (probably caused by *Ralstonia* spp., unconfirmed). This review describes global distribution, symptoms, pathogenic diversity, epidemiology and the state of the art for sustainable disease management of the major bacterial wilts currently affecting banana and enset.

## Introduction

Bananas (*Musa* spp) are the world’s most important fruit crop in terms of production volume and trade (FAOSTAT, 2014). Although a major staple in Africa, Asia and Latin America, only 13% of bananas produced are internationally traded (Lescot, 2013), indicating the fruit’s importance for domestic markets and food security. In East and Central Africa, it is a significant dietary component, ranging from about 20% of daily total food intake in Uganda up to 80% in parts of Rwanda (Abele et al., 2007). Also, the East African highland cooking bananas “Matooke” (triploid A genome East Africa group; AAA-EA) are culturally important in East Africa, with a diverse range of varieties and specific uses (Karamura et al., 1999, 2004; Kalyebara et al., 2007). In West Africa, plantains (AAB group) are grown in mixed cropping systems and play a similar role for food security and income. In Central America, cooking bananas [Bluggoe types (ABB) and/or plantains (AAB)], Gros Michel (AAA) and their dwarf mutants, and Apple (Silk, AAB), grown in mixed agroforestry systems with coffee and cocoa are an important food security crop for the rural poor in remote areas.

The Latin American and Caribbean region (LAC) accounts for 66% of global Cavendish (*Musa* AAA) exports. Ecuador is the world’s largest exporter of Cavendish bananas, with five million tons exported in 2014. The Philippines and Costa Rica are the second and third largest exporters, with 3.2 and 1.9 million tons, respectively (Lescot, 2015). In addition, LAC is also a key exporter of plantains, with 72% of plantains traded on international markets. Nevertheless, 62% of the banana and plantain production in LAC (20 million tons) is consumed locally, which indicates its high importance in diets and food security throughout the region (Dita et al., 2013).

Independent of region and production system, pests and diseases have been considered the main constraints responsible for yield losses and low productivity of bananas. The fungal diseases black leaf streak disease (commonly known as black Sigatoka), (*Mycosphaerella fijiensis*) and Fusarium wilt (*Fusarium oxysporum* f. sp. *cubense*) have always been considered as the most important banana diseases globally and have therefore received more attention. However, bacterial diseases cause significant impacts on yield globally and management practices are not always well known.

Bacterial diseases of banana and enset can be classified into three distinct groups: i) Ralstonia-associated diseases (Moko/Bugtok disease caused by *Ralstonia solanacearum* and banana blood disease caused by *R. syzygii* subsp. *celebesensis*); ii) Xanthomonas wilt of banana and enset, caused by *Xanthomonas campestris* pv. *musacearum* and iii) Erwinia-associated diseases (bacterial head rot or tip-over disease (*Erwinia carotovora* ssp. *carotovora* and *E. chrysanthemi*), bacterial rhizome and pseudostem wet rot (*Dickeya paradisiaca* formerly *E. chrysanthemi* pv. *paradisiaca*). Other bacterial diseases of less widespread importance include: Javanese vascular wilt, bacterial wilt of abaca and bacterial fingertip rot (probably caused by *Ralstonia* spp., unconfirmed).

Bacterial wilts of banana have an increasing frequency in different regions of the world reducing yield and raising crop management costs. Management practices have to be adopted according to epidemiological aspects, with site-specific and targeted actions to manage infections/eradicate infected plants and minimize pathogen spread (Blomme et al., 2014). The success of control strategies, however, strongly relies on capacity building and systematic eradication and sanitation activities. Overall, the adoption of biosafety practices at the farm and landscape level is considered as the most critical factor to manage bacterial wilts in banana after pathogen incursions are confirmed in a given production area. Blomme et al. (2014) reported on the community mobilization efforts to manage Xanthomonas wilt in East Africa. The mobilization and involvement of local communities (referred to in Uganda as Participatory Development Communication) has been an important, and possibly unique feature, of the control work on Xanthomonas wilt in East Africa. Lessons from experience with controlling Xanthomonas wilt in East Africa (both technical and social aspects) could possibly guide the management of similar bacterial wilt diseases in smallholder systems in Asia and Latin America.

**FIGURE 1:**
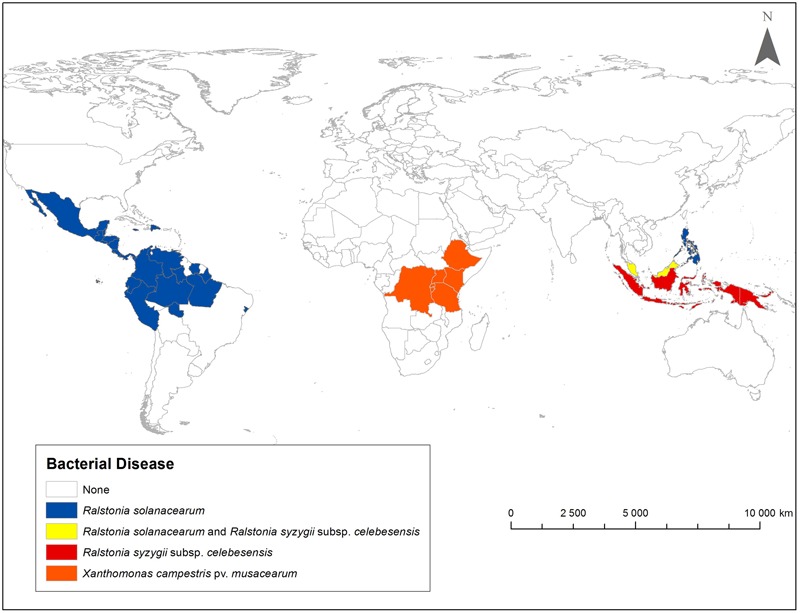
Geographic distribution of Moko/Bugtok bacterial wilt (*Ralstonia solanacearum*), blood bacterial wilt (*R. syzygii* subsp. *celebesensis*) and Xanthomonas bacterial wilt (*Xanthomonas campestris* pv. *musacearum*). Presence or absence of a disease is presented at country level.

**FIGURE 2:**
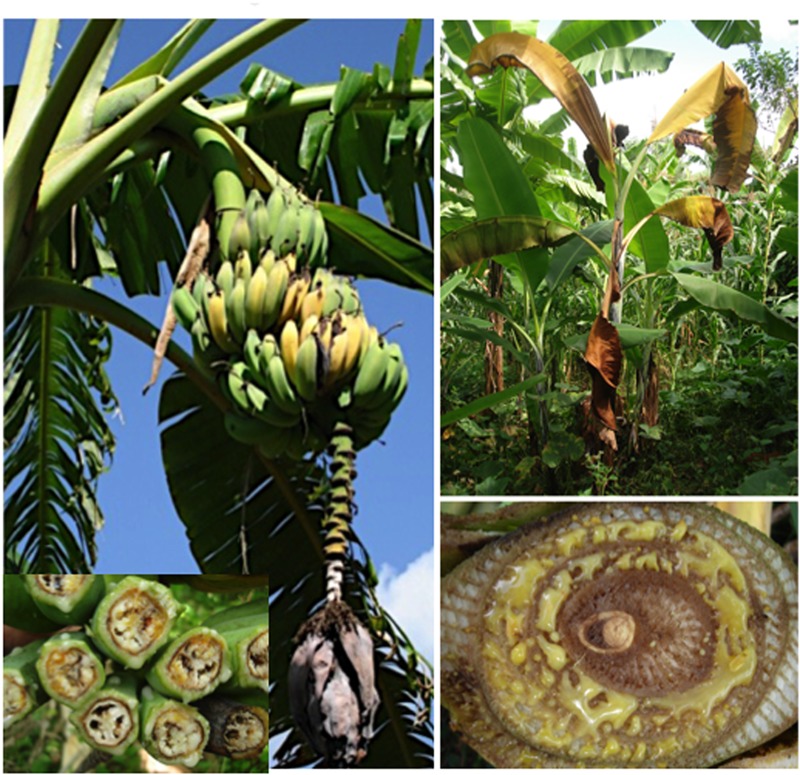
Xanthomonas bacterial wilt of banana caused by *X. campestris* pv. *musacearum*. The photos depict leaf yellowing and wilting, exudation of bacterial ooze, premature fruit ripening and fruit discoloration. Photos were taken in Uganda by Guy Blomme.

**FIGURE 3:**
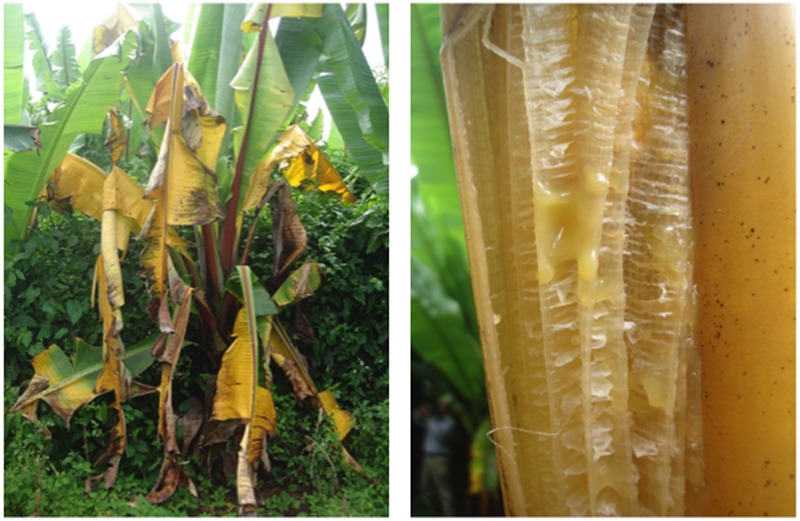
Xanthomonas bacterial wilt of enset caused by *X. campestris* pv. *musacearum*. The photos depict leaf yellowing and wilting, and pockets of bacterial ooze in a leaf petiole. Photos were taken in Ethiopia by Guy Blomme.

**FIGURE 4:**
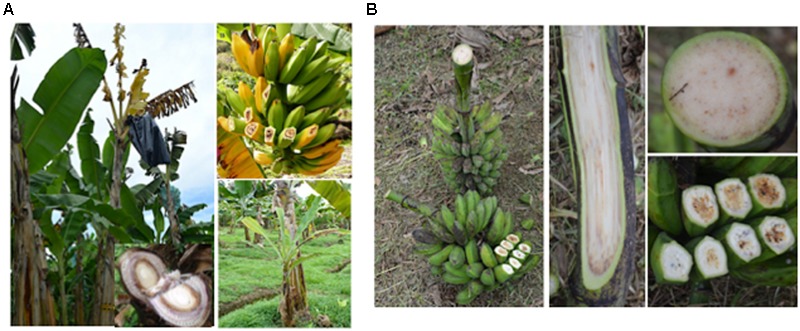
Various symptoms of Moko **(A)**/Bugtok **(B)** bacterial wilt caused by *R. solanacearum*. The photos depict (for **A**) premature fruit ripening and fruit discoloration, initial leaf symptoms on a sucker, and pseudostem discoloration; (for **B**) discoloration of fruit pulp and bunch stalk/rachis. Photos were taken in Colombia, Suriname, and Costa Rica (for Moko) and The Philippines (for Bugtok) by, respectively, Miguel Dita, Luis Pérez Vicente, and Philippe Prior.

**FIGURE 5:**
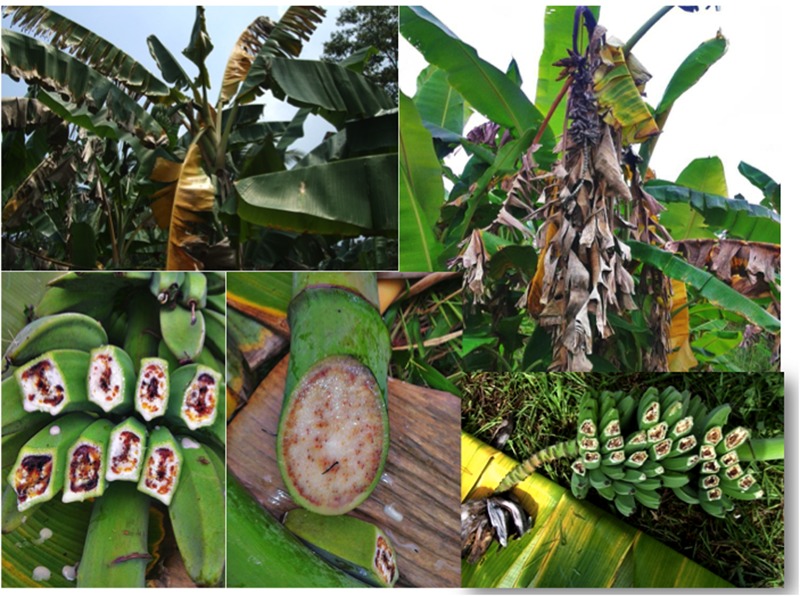
Banana blood disease caused by *R. syzygii* subsp. *celebensis*. The photos depict leaf yellowing and wilting, and fruit pulp and bunch stalk/rachis discoloration. Photos were taken in Malaysia by Agustin Molina.

**FIGURE 6:**
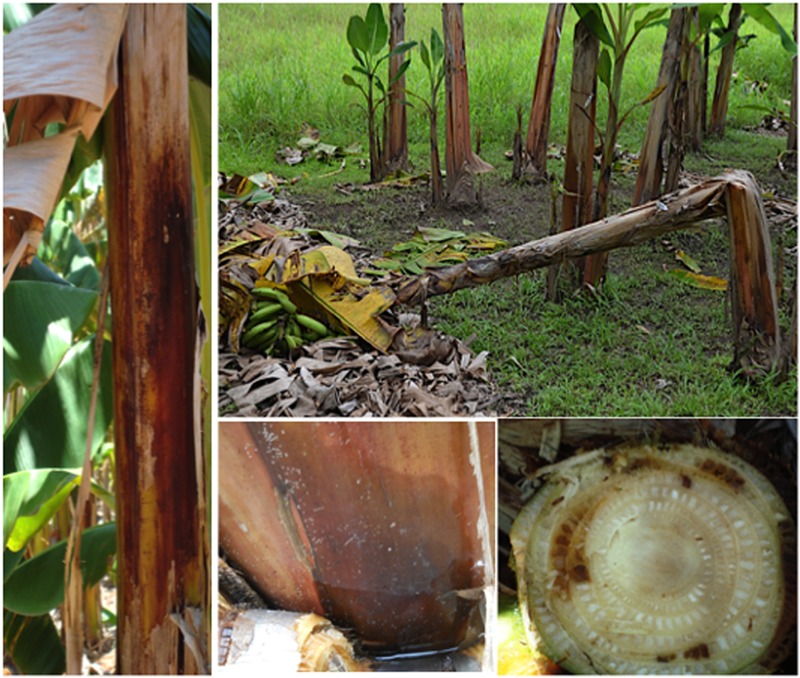
Erwinia-associated diseases (bacterial head rot or tip-over disease caused by *Erwinia carotovora* ssp. *Carotovora* and *E. chrysanthemi*, bacterial rhizome and pseudostem wet rot (*Dickeya paradisiaca* formerly *E. chrysanthemi* pv. *paradisiaca*)). The photos depict pseudostem symptoms and pseudostem doubling. Photos were taken in Panama by Miguel Dita and in Cuba by Luis Pérez Vicente.

This review describes global distribution (**Figure 1**), symptoms (**Figures 2, 3, 4A,B, 5, 6**), epidemiology and pathogenic diversity of the major bacterial wilts currently affecting bananas and enset and the state of the art for sustainable disease management.

### Causal Agents of Bacterial Wilt Diseases: Origin, Geographic Distribution, and Economic Importance

#### *Ralstonia solanacearum* Causing Moko and Bugtok Diseases

*Ralstonia solanacearum*, the causal agent of bacterial wilt, is currently found on all continents and numerous islands located between the tropics of Cancer and Capricorn, causing disease on more than 200 plant species in over 50 families (Kelman, 1953; Hayward, 1994b; Belalcazar et al., 2004). *R. solanacearum* is considered as one of the world’s most important/damaging phytopathogenic bacteria due to its lethality, broad geographic distribution and wide host range (Elphinstone, 2005; Mansfield et al., 2012). In reference to the high geographic and pathogenic diversity of the species, Buddenhagen (1986) stated that “there are many bacterial wilts and there are many ‘*Pseudomonas solanacearums’* (syn. *R. solanacearum*). They have originated and evolved in widely different places and they have different capabilities with both native flora and introduced hosts and presumably with different soils and environmental conditions.” This diversity results in variable disease expression and disease potentials for each host/parasite genotype interaction (Buddenhagen, 1986, 2009).

More than 150 years ago, Schomburgk provided the earliest reference to a bacterial wilt disease on bananas during his travels in British Guyana (since 1966 known as the independent nation of Guyana) from 1840 to 1844 (Thurston and Galindo, 1989; Sequeira, 1998). The description for Moko was published over 65 years later by Rorer (1911) following an outbreak in Trinidad, where it devastated plantations, particularly of the susceptible cultivar ‘Moko’ (*Musa* ABB, Bluggoe subgroup) from which the common name of the disease was adopted. Until the early 1950s, commercial plantations remained free of the disease, but since 1950 three consecutive bacterial wilt epidemics have swept through Central and South America, where it is now considered endemic (Sequeira, 1998; Buddenhagen, 2009). In some countries of Latin America and the Caribbean, Moko, caused by *R. solanacearum* is considered a threatening disease to bananas and plantains, together with black sigatoka (*M. fijiensis*; Lehmann-Danzinger, 1987; Sequeira, 1998). In Colombia, the disease has seriously affected the banana and plantain production and losses up to 100% in some areas have been reported (Belalcazar et al., 2004).

*Ralstonia solanacearum* has been recovered from *Heliconia* species from virgin forest in the Coto valley, southwest Costa Rica, suggesting that Moko was originally endemic in the rainforests of the Caribbean area (Sequeira and Averre, 1961). *Heliconia* species in the larger LAC, the Philippines and Indonesia, have also been known to harbor *R. solanacearum* (Elphinstone, 2005).

*Ralstonia solanacearum* strains affecting *Musa* spp. are defined by their symptom expression in the plant, cultural characteristics and whether the pathogen is spread mostly by insects or by mechanical and soil transmission (French, 1986; Sequeira, 1998). The “SFR” (small, fluidal, round) and “A” (Amazon basin) strains are known to be transmitted by insect whereas the “B” (banana) strain is transmitted through root contact and contaminated planting equipment (Sequeira, 1998). Until 1960, Moko was present in Trinidad (where it was first reported in the 1890s [Stover, 1972; Merchan-Vargas, 2003], and described by Rorer (1911), Guyana, Venezuela, Panama, Costa Rica, and Honduras. Subsequently, the “SFR” strain transmitted by insects was reported in Guatemala, El Salvador, Nicaragua, Costa Rica and Mexico (Buddenhagen and Elsasser, 1962). The occurrence of Moko in Mexico was first reported in Chiapas in 1960 (Buddenhagen, 1961) and later in Teapa and Tabasco in 1991 (Fucikovsky and Santos, 1993). The “A” strain transmitted by insects, but distinguishable in tetrazolium media, was reported later in Colombia and Peru. In this region and according to Revillo in 1967, cited by Stover (1972), Moko appeared to be disseminated along Peruvian Amazon tributary rivers.

The current distribution of Moko in Latin America covers the following countries: Belize, Brazil (in the Amazon basin and in Sergipe), Colombia, Costa Rica, Ecuador, El Salvador, Grenada, Grenadines, Guatemala, Guyana, Honduras, Jamaica, Mexico, Nicaragua, Panama, Peru, St. Vincent, Suriname, Trinidad and Tobago and Venezuela (CAB International, 2014). A previous report by the European Plant Protection Organization (EPPO) of Moko in Cuba is unreliable due to the fact that there has not been an outbreak of Moko in the last 45 years in this country and existing *R. solanacearum* reports are related to a geographically restricted distribution of race 1 (not covering strains pathogenic to banana) in tomatoes introduced with potato seeds from Europe.

Moko is also present in the Philippines (southern Mindanao) and could have been introduced there through infected ‘Valery’ (*Musa* AAA Cavendish subgroup) planting materials (Rillo, 1979; Buddenhagen, 1994). Yet the origin of this disease in the Philippines remains disputed, as there is reason to believe that strains of the disease were already present on solanaceous and other crops, including *Musa textilis* in 1969 (Zehr, 1970; Eden-Green, 1994a; Seal and Elphinstone, 1994; Taghavi et al., 1996). A first report of *R. solanacearum* causing Moko disease of banana in Malaysia was published by Zulperi and Sijam (2014). Reports of the occurence of banana bacterial wilt in Cambodia and India have not been confirmed (Jones, 2000; Daniells, 2011). An outbreak of *R. solanacearum* on ornamental *Heliconia* spp. in Australia was eradicated (Jones, 2000).

The name “Bugtok” is used when wilt symptoms, caused by *R. solanacearum*, appear on ABB cooking type bananas in the Philippines (Molina, 1999, 2006). It was a very serious disease of Saba (*Musa* BBB) in the early 1990s. The major transmission of this pathogen on Saba is by insects (Molina, 1999, 2006). Tool transmission seems less frequent most likely as a result of less intensive management. Through simple early male bud removal, the disease has been managed to acceptable levels in both medium-scale farms (for banana chips production) and subsistence farms. Early de-budding has now become a cultural practice among farmers not only to control the disease but also to use the male bud as a vegetable. The same pathogen causes “Moko” in large-scale Cavendish plantations (Molina, 1999, 2006). Transmission in the large-scale Cavendish plantations is mainly through pruning tools. Insect transmission is very rare in Cavendish because bunches are bagged with plastic at the time of shooting to prevent insect damage and possible insect-mediated disease transmission (Stover and Simmonds, 1987).

The causal agent of Bugtok in the Philippines has been shown to be identical to the “B” strain of *R. solanacearum* from Honduras, as both strains are indistinguishable by numerical taxonomy or genetic analyses (Eden-Green, 1994a; Thwaites et al., 1999; Ilagan et al., 2003; Raymundo et al., 2005). Furthermore, the low genetic diversity among *R. solanacearum* strains isolated in the Philippines from *Musa* spp. (Raymundo et al., 1998, 2005), suggests that a single genotype of the pathogen was introduced from Central America.

#### *Ralstonia syzygii* Subsp. *celebesensis* Causing Banana Blood Disease

Banana blood disease is thought to have originated on Salayar Island near Sulawesi, where it was first reported after the introduction of dessert bananas in the early 1900s (Eden-Green, 1994b; Thwaites et al., 2000). The disease was confined to Salayar for many years due to the strict quarantine regulations implemented by the Dutch. However, it had become widespread on local cooking banana cultivars in southern Sulawesi (formerly Celebes) by 1920 (Gäumann, 1921; Stover and Espinoza, 1992), and then probably spread throughout the island until its discovery in Java in the late 1980s (Thwaites et al., 2000).

Unfortunately, the pathogen has since continued its spread to most of the larger Indonesian islands, where average yield losses often exceed 35% (Supriadi, 2005). These outbreaks were associated with the transmigration of people from Java to less populated islands in Indonesia (Ploetz et al., 2015). The banana blood disease is currently spreading in peninsular Malaysia where it coexists with the Moko and Fusarium wilt diseases (Teng et al., 2016). The disease has been first detected in the province of Perak and more recently in the province of Selangor (Heng, 2012; Kogeethavani et al., 2013; Teng et al., 2016). Banana blood disease has also been observed on the island of New Guinea (Davis et al., 2001). Severe destruction due to banana blood disease was noted in South Sulawesi, where 70–80% of plantations were lost (Roesmiyanto and Hutagalung, 1989), and in West Java, where 27–36% plantation loss was recorded (Subijanto, 1991). In Lampung Province (Sumatra), more than 20,000 tons of banana, with an estimated value of US$1 million, were lost to banana blood disease (Nurhadi and dan Harlion, 1994). Losses will most likely escalate with disease spread. If the disease 1 day arrives on the South-East Asian mainland there would be no barriers to its eventual/gradual movement to the Indian subcontinent (Jones, 2013; Ploetz et al., 2015).

#### *Xanthomonas campestris* pv. *musacearum* Causing Xanthomonas Wilt of Banana and Enset

Caused by the bacterium *X. campestris* pv. *musacearum* (*Xcm*), XW symptoms were first observed on enset in Ethiopia in the 1930s (Castellani, 1939). The disease was, however, first identified in Ethiopia as Xanthomonas wilt in 1968 on enset (Yirgou and Bradbury, 1968) and subsequently on banana in 1974 (Yirgou and Bradbury, 1974). Since 2001, it has been reported from Uganda (Tushemereirwe et al., 2003), eastern Democratic Republic of Congo (DR Congo) (Ndungo et al., 2004), Rwanda, Tanzania (Mgenzi et al., 2006; Carter et al., 2010), Kenya (Mbaka et al., 2009; Carter et al., 2010) and Burundi (Carter et al., 2010). In DR Congo, Xanthomonas wilt has also been reported in recent years, by the provincial agricultural research and extension services, in Uvira and Fizi in South Kivu province, in the Kalemie territory of northern Katanga province, and in Tshopo district in Oriental province (Anonymous, 2012). Since 2001, Xanthomonas wilt has become the most important and widespread disease of *Musa* in East and Central Africa. Its non-discriminate infection of all *Musa* cultivars in this region (Ssekiwoko et al., 2006c) and ability to cause up to 100% yield loss especially in ABB type bananas, severely compromises food security and livelihoods for banana-based farming households (Tushemereirwe et al., 2003, 2004; Kagezi et al., 2006; Ssekiwoko et al., 2006a,b). In fact, low soil fertility and Xanthomonas wilt are currently considered as the two principal threats to banana productivity in the East African Great Lakes region (Kalyebara et al., 2007).

#### *Dickeya paradisiaca* Causing Pseudostem and Rhizome Rot

Pseudostem wet rot was first reported in the Cauca valley of Colombia (Llanos, 1967; Fernández and López, 1970), where it caused serious losses in nearly 2000 hectares of plantains. The disease is widely distributed in plantain and banana in Guatemala (Wardlaw, 1972), Cuba (Rivera, 1978), Jamaica (Shillingford, 1974), Haiti, Venezuela (Ordosgoitti et al., 1974), Colombia (Fernández, 1967); Ecuador and Peru and Nicaragua, Panama and Dominican Republic (Dita et al., 2013). In the 1970s, the disease caused serious damage in plantains in Cuba, with incidence in some fields of up to 75%. Currently, the disease seriously affects plantations of plantain in El Salvador, Nicaragua, Panama and Dominican Republic (Dita et al., 2013), where losses up to 50% were informally reported.

#### *Pectobacterium carotovorum* Causing Bacterial Head Rot or Rhizome Rot

Pectobacterium head rot is a common disease of banana and plantain in the humid tropics that causes a soft rot of the rhizome of banana and plantain plants growing in cool damp humid soils or in suckers (Stover, 1972; Buddenhagen, 1994). Infected plants are commonly observed after heavy rainfall periods in soils with poor drainage.

#### Banana Wilt Disease Associated Phytoplasmas in Papua New Guinea

Plants with distinct disease symptoms, clearly different from those produced by Fusarium or bacterial wilts, were observed during bacterial wilt surveys in ABB cooking banana fields in Papua New Guinea. These surveys were carried out by the Papua New Guinea National Agricultural Quarantine and Inspection Authority (NAQIA) and Australia’s Department of Agriculture, Fisheries and Forestry (DAFF) from 2008 to 2012 (Davis et al., 2012). Subsequent diagnostic studies revealed the presence of phytoplasmas related to the coconut lethal yellowing disease phytoplasma group.

### Bacterial Disease Symptoms: Communalities and Differences

Symptoms of diseases caused by bacteria in banana can be summarized as wilting, plant toppling and rotting of rhizome, pseudostem and/or fruits.

Wilting starts when pathogen densities increase throughout the plant, which prevents sufficient water from reaching the leaves due to vascular dysfunction (Buddenhagen and Kelman, 1964; Denny et al., 1990). The process by which colonization by bacterial wilts reduces water flow is not completely clear. There is no evidence for excessive transpiration linked to loss of stomatal control as could possibly result from a systemic toxin (Buddenhagen and Kelman, 1964; Van Alfen, 1989). The primary factor is most likely plugging of pit membranes in the petioles and leaves by a high molecular mass extracellular polysaccharide (Van Alfen, 1989), but high bacterial cell densities, plant-produced tyloses and gums, and byproducts of plant cell wall degradation may be contributing factors (Denny, 2006).

A study by Minguez et al. (2011) on the histological and morphological characterization of ‘Cardaba’ (ABB) and ‘Cavendish’ (AAA) roots infected by Moko and Bugtok pathogens confirmed that bacteria colonized and degraded the cell walls of the xylem vessels and intracellular spaces, particularly in protoxylem vessels. The Bugtok isolate was more aggressive than Moko, which showed poor invasion capacity. Although tylose formation was also found, results suggested that wilting was not only due to bacterial occlusion but also due to the destruction of cell walls of xylem vessels (Minguez et al., 2011). Wilting may be observed on plants infected by *R. solanacearum* and *X. campestris* pv. *musacearum* (**Figures 2, 3, 4A,B, 5**).

Typical Moko wilt symptoms appear once the pathogen has systemically colonized the pseudostem and underground rhizome. Infected dessert banana plants exhibit rapid yellowing and wilting of leaves and physically attached suckers, vascular discoloration in the pseudostem leafsheaths, premature fruit ripening or arrested fruit development and fruit blackening, and dry rot of fruit pulp (Thwaites et al., 2000; Denny, 2006). Bacterial ooze can be readily observed in internal tissues of any part of the plant that becomes exposed to the air. In certain conditions internal pseudostem discoloration caused by *R. solanacearum* (Moko) can be confused with Fusarium wilt and *in loco* diagnosis needs to be done by experts. The inspection of bunches to observe rotting fruits, the presence of young distorted rotting suckers and bacterial oozing from exposed tissues is a common practice to discriminate between Moko disease (**Figure 4A**) and Fusarium wilt as rotting fruit and bacterial ooze do not appear in plants with Fusarium wilt.

Differences in inflorescence morphology across cultivars results in varying degrees of susceptibility to (insect-mediated) infection by bacterial wilts. Host-pathogen interaction and the importance of cultivar susceptibility and management practices on symptom development are illustrated by Bugtok in the Philippines and the B strain of Moko from Honduras.

In the Philippines, Moko and Bugtok are two names describing different symptoms of the same disease caused by the same *R. solanacearum* strains. The symptoms are different because of a difference in epidemiology brought about by contrasting variety-cropping systems (Molina, 1999). Moko is the term used for leaf wilting and yellowing symptoms observed in medium- to large-scale Cavendish plantations, while Bugtok describes fruit-rotting symptoms mainly observed in balbisiana cultivars (i.e., Saba a BBB cooking banana grown for local markets (Molina, 2006). In commercial plantations of Cavendish dessert bananas in the Philippines, the Bugtok strains show similar symptom development to the Moko B strain from Honduras. In smallholder farms in the Philippines, on the local cooking banana varieties ‘Saba’ (*Musa* BBB), ‘Cardaba’ (*Musa* ABB) and ‘Latundan’ (*Musa* AAB), Bugtok symptoms are limited to the inflorescence, with the rachis/peduncle becoming black, dry and often distorted. In addition, fruit pulp becomes discolored grayish black to yellowish red and later becomes hard. There may also be a reddish brown discoloration of the vascular tissue of the pseudostem and peduncle, but rarely does this discoloration extend into the underground rhizome. Because the pathogen is never fully systemic, there are no leaf yellowing or wilting symptoms in Bugtok and the plant appears relatively normal/healthy to the untrained eye (Denny, 2006; Molina, 2006).

Bugtok is a result of inoculation by insects vectors through the male flowers, thus symptoms of rotting occur first in the fruits. Moko symptoms mainly occur when inoculation starts from the basal part of the plant, usually through contaminated tools used during pruning and de-suckering, a common practice in Cavendish cropping systems. Moreover, commercial Cavendish plantations practice early male flower removal and bagging of bunches with plastic bags to protect them from insect transmission (Molina, 2006). Subsistent to medium-scale Saba growers do not practice de-suckering or pruning thus “Moko” symptoms are very rare. Moreover, growers do not cover the fruits with plastic bags and early de-budding is rarely practiced.

In 1992, an artificial inoculation experiment was carried out in Honduras using the Moko pathogen. The Cavendish cultivar ‘Grand Naine’ was inoculated at the inflorescence level and in roots, this to, respectively, simulate insect-vectored transmission versus mechanical (i.e., pruning, de-suckering) or even soil-root inoculation. Root-inoculated plants showed typical Moko symptoms (i.e., leaf yellowing and wilting), while inflorescence-inoculated plants showed typical Bugtok symptoms (inflorescence wilting). This experiment showed that for the same pathogen, symptom development is linked to infection court.

Symptoms of *Xanthomonas* wilt do not differ markedly from *R. solanacearum.* The incubation period for *Xanthomonas* wilt is about 3 weeks and, as for *R. solanacearum*, depends on cultivar, plant growth stage, mode of disease transmission (i.e., through an infection court on the male infloresence part or on the pseudostem or leaves) and environmental conditions (Mwangi et al., 2006; Ssekiwoko et al., 2006a,b; Tripathi et al., 2008; Tripathi and Tripathi, 2009; Addis et al., 2010; Ocimati et al., 2013a).

Visible Xanthomonas wilt symptoms after an insect-mediated infection on the male inflorescence part include wilting of male bud bracts, followed by drying of the rachis coupled with bacterial exudation, often followed by premature ripening of some or all of the fruits, and eventually wilting and death of the entire plant (Ssekiwoko et al., 2010; Ocimati et al., 2013a,b,c; Nakato et al., 2014). An internal cross-section of a floral stalk shows yellow bacterial ooze from the vascular bundles, while a cross section of a fruit shows rusty brown stains in the fruit pulp (Thwaites et al., 2000; Tushemereirwe et al., 2003; Biruma et al., 2007; Ocimati et al., 2013a,b). Late floral symptoms (when the banana bunch is physiologically mature) have also been reported due to tool-mediated infections (Ocimati et al., 2013a). Xanthomonas wilt bacteria entering the corm, roots, pseudostem and leaves of banana plants, e.g., through garden tool use, will first cause a progressive yellowing and wilting of the leaves (Tushemereirwe et al., 2004; Ssekiwoko et al., 2006b; Ocimati et al., 2013a,b,c; Nakato et al., 2014). In addition, a yellow- or cream-colored ooze, typical of many bacterial infections, exudes within a couple of minutes of cutting tissue of an infected plant, and copious quantities of ooze may be produced over a period of several hours. A cross-section of a diseased pseudostem reveals brown or yellow streaks in the vascular tissue and yellowish bacterial ooze (Tripathi, 2005). The affected pseudostems most often wilt and die.

Symptoms of banana blood disease share many characteristics with the insect-transmitted “A” and “SFR” strains of *R. solanacearum* causing Moko disease, namely discoloration and shriveling of the male flower bud and peduncle, reddish dry rot of the fruit pulp and reddish discoloration of vascular tissue throughout the plant, which emits reddish-brown bacterial ooze when cut. Older leaves become yellow, followed by wilting, necrosis and collapse; younger leaves turn bright yellow before becoming necrotic and dry. The pathogen rapidly colonizes the entire plant, and suckers will also wilt and die (Eden-Green and Seal, 1993; Eden-Green, 1994b; Supriadi, 2005). *R. syzygii* subsp. *celebesensis* strains causing banana blood disease is strictly related to banana (and some *Heliconia*) and not to Solanaceaous hosts, unlike *R. solanacearum* strains causing Moko/Bugtok disease.

Pseudostem wet rot and rhizome rot caused by *D. paradisiaca* are attributed to the proteolytic enzymes it produces. Pseudostem wet rot symptoms initially appear as translucid spots on sheaths in different parts of the pseudostem or in the base of leaves. Later they become reddish brown, to finally take a dark brown color and cover a large part of pseudostem. The rot advances down in the pseudostem and toward the interior of leaf sheaths and stops when it reaches the bunch stalk. A fetid, amber-color liquid emerges when pressure is applied to affected tissues. Severely infected plants can develop young chlorotic leaves with necrotic margins and dwarf buds. Severely affected young plants do not flower (Stover, 1972; Rivera, 1978). An additional symptom mostly observed in ‘Cavendish’ (AAA) plants is a cream to dark brown sheath rot at soil level that later evolves into a necrotic cavern in the rhizome (Rivera and Ezavin, 1980) resembling those caused by *Cosmopolites sordidus*. Plants developing from infected rhizomes show slow growing, chlorotic and flaccid leaves, as well as a rotting that spreads upward from the pseudostem base to the rest of the pseudostem (Rivera and Ezavin, 1980). These plants may eventually collapse and die. Weakened plants can fall down easily and break at soil level. Infected planting material develops weak buds and shoots that are often destroyed by the ascendant rot from the rhizome. In heavily infected fields, plant doubling (i.e., pseudostem breakage) is commonly observed and this phenomenon may be accelerated by wind and bunch weight. In contrast to toppling/snapping caused by, respectively, nematodes and weevils, where the root system/corm is exposed, bacteria-associated doubling occurs at some distance above the base of the pseudostem (**Figure 6**).

Plants affected by *Pectobacterium carotovorum* show rotting with poor sprout emergence, dwarfing, yellowing and wilting of leaves, slow and retarded growth of plants and toppling over of mature plants and fruits (Stover, 1972). The rhizome cortex shows soft humid brown to cream pockets that enlarge covering most of rhizome cortex.

#### Phytoplasma Wilt Disease Symptoms

Phytoplasma wilt external symptoms consist of yellowing and leaf death, meanwhile inside pseudostems, discontinuous streaking appears as small sections of black or brown vascular tissues, usually with wet and necrotic pockets (Davis et al., 2012).

### Taxonomy and Genetic Diversity of Causal Agents

**Table 1** gives an overview of taxonomic classifications used to identify the major bacterial diseases affecting banana and enset, including the most recent genetic, geographic and ecotype diversity (Eden-Green, 2006; Genin and Denny, 2012).

**Table 1:** Bacterial wilts affecting *Musa* spp.

Common name	Distribution and hosts	Traditional taxonomy	Currently accepted taxonomy	Proposed species^a^
			Phylotype/sequevar	
Moko	America (Mexico), Venezuela, Guyana, Colombia, Peru; Brazil; Caribbean (Grenada, Dominican Republic and Jamaica; all cultivated banana types and wild *Heliconia*), Philippines (AAA types; “Moko”); Malaysia	*Ralstonia solanacearum* biovar 1, race 2	IIA-6, IIA-24, IIA-41, IIA-53, IIB-3, IIB-4, and IIB-25	*Ralstonia solanacearum*

Bugtok, Tibaglon, Tapurok	Philippines (on ABB/BBB types; “Bugtok”)	*Ralstonia solanacearum* biovar 1, race 2	IIB-3	*Ralstonia solanacearum*

Banana blood disease, Penyakit darah	Indonesia (all cultivated types; some wild types may be resistant), Malaysia and Papua New Guinea.	Banana blood disease bacterium (*Ralstonia* spp.)	IV-10	*Ralstonia syzygii* subsp. *celebesensis*

Xanthomonas bacterial wilt of banana and enset (enset wilt, banana bacterial wilt)	Ethiopia, Uganda, DR Congo, Rwanda, Burundi, Tanzania, Kenya (enset and all cultivated banana types).	*Xanthomonas campestris* pv *musacearum* (*Xcm*)	Not relevant	*Xanthomonas vasicola* pv. *musacearum*

*^a^Proposed species: Aritua et al. (2008), Safni et al. (2014).*

#### The *Ralstonia solanacearum* Species Complex

*Ralstonia solanacearum* was first described and classified as *Bacterium solanacearum* by Erwin F. Smith at the end of the 19th century (Smith, 1896). The causal agent of bacterial wilt was then successively named *P.* s*olanacearum*, and more recently, by application of DNA-based methods, *Burkholderia solanacearum* (Yabuuchi et al., 1992) and finally *R. solanacearum* (Yabuuchi et al., 1995).

The genus *Ralstonia* belongs to the family *Burkholderia* (class Betaproteobacteria) that includes nine genera and many human- and plant-pathogenic species and several symbionts. *Ralstonia* is an aerobic, Gram-negative rod with a polar flagella tuft. It is oxidase positive, arginine dihydrolase negative, and accumulates poly-hydroxybutyrate intracellularly. Most strains denitrify and produce a diffusible brown-red pigment on rich medium. It does not grow below 4°C or above 40°C, and there is little or no growth in 2% NaCl (Baharuddin et al., 1994; Taghavi et al., 1996; Coenye and Vandamme, 2003; Villa et al., 2005).

*Ralstonia solanacearum* is a heterogeneous species, as demonstrated by its large host range, pathogenic specialization and physiological and cultural properties, as well as its phylogeny (Hayward, 1991). Despite being classified as a single species, it has been reported that different strains of *R. solanacearum* may have DNA–DNA relatedness values below 70% (Palleroni and Doudoroff, 1971) and therefore could possibly be members of different species. The term ‘species complex,’ which refers to ‘a cluster of closely related bacteria whose individual members may represent more than one species,’ was then proposed for *R. solanacearum* (Gillings and Fahy, 1994). Recently, Safni et al. (2014) provided evidence, reported below, for three different species, thereby justifying the use of the term “species complex.”

It is assumed that *R. solanacearum* originated, adapted and evolved in widely different places, resulting in great geographic and pathogenic diversity and translating in variable disease expression and disease potentials for each host/parasite genotype interaction (Buddenhagen, 1986, 2009). However, recent studies suggest that *R. solanacearum* most likely originated in Oceania/Indonesia, and migrated to Africa, South America and Asia, possibly before the fragmentation of the ancestral continent Gondwana (Remenant et al., 2011; Wicker et al., 2012).

Traditionally, strains of *R. solanacearum* were divided using the terms “race,” “strains,” and “biovar,” based on pathogenicity and biochemical characteristics. The “biovar” classification system, originally proposed by Hayward (1964), groups strains identified as *R. solanacearum* according to their ability to metabolize specific substances, i.e., to acidify media containing specific carbohydrates, to produce nitrite from nitrate and to produce gas from nitrate (Hayward, 1994a; Denny and Hayward, 2001). Following this traditional classification system, the causal agent of Moko (and Bugtok) disease was recognized as *R. solanacearum* race 2, biovar 1 (Buddenhagen et al., 1962; Buddenhagen and Kelman, 1964; Buddenhagen, 1986; Soguilon et al., 1995; Eyres et al., 2005). Although useful, the biovar system lacks discriminating power due to its limited genetic basis (Denny, 2006). With regards to the “race” concept, several authors have recognized that the “races” of *R. solanacearum* in fact resemble pathovars, as is common for other species of phytopathogenic bacteria (Alvarez, 2005). A pathovar is a subspecific division that groups all bacterial strains that cause the same symptoms on the same plant host range (Dye et al., 1980; Schaad, 1987).

The race-biovar system has now, in most cases, been replaced by the widely accepted phylotype-sequevar hierarchical classification scheme. Phylotypes are defined as a monophyletic cluster of strains revealed by phylogenetic analysis of sequence data. Phylotypes are therefore major phylogenetic subdivisions within the *R. solanacearum* species complex and sequevars are clusters of strains whose endoglucanase (*egl*) partial sequences differ by less than 1% (Fegan and Prior, 2005; Genin and Denny, 2012). More recently, Wicker et al. (2012) used multilocus sequence analysis (MLSA) to retrace evolutionary history within the *R. solanacearum* species complex. Using sequences of seven housekeeping genes (*gdhA, mutS, ppsA, adk, leuS, rplB, gyrB*) and two virulence-associated genes (*fliC* and *egl*), eight clades comprising strains with distinct evolutionary patterns were identified (Wicker et al., 2012). The *R. solanacearum* species complex is subdivided into four distinct phylotypes, largely correlating with the geographic origin and evolutionary past of the strains (**Table 1**) Strains are assigned to the Asian (phylotype I), American (II), African (III), and Indonesian (IV) phylotypes (Fegan, 2005; Prior and Fegan, 2005; Fegan and Prior, 2006). Phylotype IV hosts the two closely related bacteria *R. syzygii* (the causal agent of Sumatra disease of clove) and the ‘blood disease bacterium (BDB)’ (Seal et al., 1993; Vaneechoutte et al., 2004; Fegan and Prior, 2005; Villa et al., 2005; Remenant et al., 2011).

Using a polyphasic taxonomic approach, Safni et al. (2014) proposed to merge the *R. solanacearum* species complex into three species: *R. solanacearum* corresponding to phylotype II strains (including Moko strains); *R. pseudosolanacearum* corresponding to phylotypes I and III; and *R. syzygii* corresponding to phylotype IV. *R. syzygii* sp. nov is further divided into three subspecies: the broad host range strains are *R. syzygii* subsp. *indonesiensis* subsp. nov.; the strains causing Sumatra disease of cloves as *R. syzygii* subsp. *syzygii* subsp. nov.; and the BDB strains causing the banana blood disease as *R. syzygii* subsp. *celebesensis* subsp. nov. Comparative analysis of 29 whole genomes by MUMi and the use of protein profiling on a larger set of bacterial strains by matrix-assisted laser desorption/ionization-time of flight mass spectrometry (MALDI-TOF-MS), support the division of the *R. solanacearum* species complex into three species consistent with genomic and proteomic data as well as biological differences (Prior et al., 2016).

The polyphyletic nature of the *R. solanacearum* (phylotype II) strains causing the Moko disease remains to be elucidated by an in-depth comparison of genomes. In recent literature it has been suggested that pathogenicity to banana lies in a very restricted number of genes (or even allelic forms of the same genes) that may be easily transferable through horizontal gene transfer (Wicker et al., 2012). Although elegant, this assumption was not supported by recent comparative genomic work (Ailloud et al., 2015). The robustness of the phylotype classification, thus far, would imply that it reflects true evolutionary lineages within the *R. solanacearum* species complex. These lineages presumably developed when progenitors became geographically isolated and subsequently adapted to different environments and potential host plants (Denny, 2006). Isolates studied in Brazil from banana and *Heliconia* belong to phylotype II and sequevars IIA-6, IIA-24, IIA41, IIB-25, and to a new sequevar IIA53 (Albuquerque et al., 2014), showing the variability of the pathogen in Brazil. The results support the effectiveness of the *egl* gene in revealing relationships among strains.

*Ralstonia syzygii* subsp. *celebesensis* (BDB) was historically described and named *P. celebensis* in 1921, but the name became invalid when the original type strain was lost (Gäumann, 1921; Eden-Green, 1994b). Jones (2000) suggested that the blood disease pathogen coevolved with banana. Buddenhagen (2009) however, indicated that this was unlikely due to differences in when and where the disease first appeared. Blood disease was first observed where wild bananas were not found (Rijks, 1916), supporting the suggestion that the bacterium originated on other plant species than banana (Buddenhagen, 2009). Colonies of the ‘banana blood disease’ strains are smaller than those of *R. solanacearum* causing Moko and are slow-growing, non-fluidal on Kelman’s TZC (Triphenyl Tetrazolium Chloride) medium (commonly used for *R. solanacearum*) and have smooth margins with a dark-red center (CAB International, 2014). Genetic analyses, by whole genome RFLP groupings, comparison of partial 16s ribosomal DNA sequences and analysis of tRNA consensus primer amplification products, indicate a close relationship, but distinctly different from other strains of the *R. solanacearum* species complex (Seal et al., 1993; Eden-Green, 1994a,b). Genetic analyses revealed that there is little diversity among strains of BDB (Thwaites et al., 1999; Fegan and Prior, 2006), suggesting few introductory and founder events as well as a recent evolutionary past on banana (Ploetz et al., 2015).

#### *Xanthomonas campestris* pv. *musacearum*

*Xanthomonas* is a genus within the Gammaproteobacteria that includes 420 species and hundreds of pathovars of Gram-negative, rod-shaped, plant-pathogenic bacteria (Vauterin et al., 1995). The genus affects at least 44 plant families, including some economically important ones such as *Solanaceae, Leguminosae*, and *Zingiberaceae* (Biruma et al., 2007). Historically, many of these pathovars were created following the 1980 publication of bacterial names by the International Committee on Systematic Bacteriology, wherein many bacterial species were deemed inadequately described and thus reduced to the level of ‘pathovar.’ Within the genus *Xanthomonas*, four species were retained out of the 30 or more then-recognized species. The 26 other species were reduced to the pathovar-level and included in the type species *X. campestris* (Schaad, 1987).

The causal agent of Xanthomonas wilt of banana was originally described as *X. musacearum* (Yirgou and Bradbury, 1968) and subsequently classified as *X. campestris* pv. *musacearum* (*Xcm*) (Young et al., 1978). Aritua et al. (2008) showed that strains of *X. campestris* pv. *musacearum* are homogeneous and very similar to *X. vasicola*. Therefore, *Xcm* was suggested (but not yet formally accepted) as a new pathovar of species *X. vasicola* pv. *musacearum* (*Xvm*) (Aritua et al., 2008). *X. vasicola* includes causative agents of several economically important diseases, including the pathovars *X. vasicola* pv. *holcicola* (Xvh) pathogenic to sorghum, and *X. vasicola* pv. *vasculorum* (Xvv) pathogenic to sugarcane (*Saccharum officinarum*) and maize (*Zea mays*) (Ohobela and Claflin, 1987; Vauterin et al., 1992, 1995). Complete genome sequences have been reported for several members of Xanthomonas (da Silva et al., 2002; Lee et al., 2005; Pieretti et al., 2009; Moreira et al., 2010). Although a genome sequence is available for *X. vasicola* strain NCPPB4381, little is known about the molecular genetics and ecology of the banana pathogen Xcm or its close relatives Xvv and Xvh (Studholme et al., 2010). Aritua et al. (2008) demonstrated that strains of Xvh and Xvv were non-pathogenic on banana but were pathogenic on maize, whereas Xcm strains were pathogenic on both banana and maize. These pathogenicity data suggest a host-jump by a strain of Xvh or Xvv onto a *Musa* species, because the Xcm strains retained pathogenicity to maize (Aritua et al., 2008).

Wasukira et al. (2012) used genome-wide sequencing to discover a set of single-nucleotide polymorphisms among multiple East African isolates of *Xcm*. Their analysis revealed two major sub-lineages of the pathogen, suggesting that current outbreaks of Xanthomonas wilt on *Musa* species in the East African Great Lakes region may have had more than one introductory event, perhaps from Ethiopia (Wasukira et al., 2012).

In addition and based on comparisons of genome-wide sequence data from multiple isolates of Xcm and multiple strains of *X. vasicola* pathovar *vasculorum*, Wasukira et al. (2012) identified genes specific to Xcm that could be used to specifically detect Xcm by PCR-based methods.

Karamura et al. (2015) carried out large-scale comparative pathogenicity studies using *X. vasicola* strains and *X. campestris* pv. *musacearum* strains on banana, maize and sugarcane. The six strains of *X. campestris* pv. *musacearum* used in the experiments caused disease in sugarcane and banana but not on maize. Two and four strains of *X. vasicola* pv. *vasculorum* and *X. vasicola* pv. *holcicola*, respectively, were not only pathogenic on maize and sugarcane but each also caused distinct symptoms on maize. *X. vasicola* pathovar *vasculorum* caused deformation of the maize plants while *X. vasicola* pathovar *holcicola* caused stunted growth.

#### *Dickeya paradisiaca* and *Pectobacterium carotovorum*

Bacteria associated with banana soft rots have been described as *D. paradisiaca* (previously *E. chrysanthemi*; Dickey and Victoria, 1980; Samson et al., 2004) and *P. carotovorum*.

*Dickeya paradisiaca* belongs to Enterobacteriaceae (class Gammaproteobacteria) and is an aerobic, Gram negative rod, with peritrichous flagella, that appears single or in pairs that do not form spores. It is protopectinase, amylase, nitrate reductase, lecitinase and catalase positive; amylase, urease, oxidase, and gelatinase negative and produces gas from glucose. It does not grow at 5% NaCl but can grow at 40°C (Rivera, 1978; Rivera and Ezavin, 1980). Colonies in nutrient agar after 48 h are white to light gray, have irregular borders, fine granular growth, and after 4 days show a well-defined rising center. In YDC (Yeast extract-Dextrose-Calcium Carbonate) media (Dye, 1969) some isolates may produce a blue non-diffusible pigment. In King B media (King et al., 1954) it produces a brown diffusible pigment. The pathogen can be selectively isolated with MNL culture media (Hevesi et al., 1981).

Data on genetic diversity of *D. paradisiaca* are extremely scarce and more studies should be conducted as the disease is increasing and, in some circumstances, the causal agent is not always clear. In artificial inoculation studies, Rivera and Ezavin (1980) and Rivera et al. (1980) found that *D. paradisiaca* isolates obtained from necrotic rhizome rot and pseudostem wet rot differ in pathogenicity and aggressiveness. Isolates from necrotic rhizome can infect and cause lesions in rhizomes cortex and pseudostem tissues. However, isolates recovered from pseudostem wet rot lesions are only able to affect leaf sheaths, but not the rhizomes cortex. Plants developing from infected rhizomes show slow growing, chlorotic and flaccid leaves, as well as a rotting that spreads upward from the base of the plant to the rest of the pseudostem (Rivera and Ezavin, 1980). These plants may eventually collapse and die (**Figure 6**). *P. carotovorum* (formerly *E. carotovora*) belongs to Enterobacteriaceae (class Gammaproteobacteria) and is an aerobic, Gram negative rod, with peritrichous flagella, does not form spores, and produces grayish white colonies on nutrient agar. This organims has been mainly associated with head rot in banana.

#### Phytoplasma Wilt Disease in Papua New Guinea

Nested PCR diagnostic analysis of vascular samples of wilted banana plants in Papua New Guinea were positive when using 16S rDNA internal control primers (Davis et al., 2012). The 16S rRNA gene, 16S-23S spacer region and a part of the 23S rRNA gene and the ribosomal protein (rp) S19 (rps19), ribosomal protein L22 (rpl22), and ribosomal protein S3 (rps3) genes of phytoplasmas from samples, were amplified using the P1/P7 and rpL2F3/rp(I)R1A primer pair (according to Martini et al., 2007), respectively. Sequences of 16S rRNA gene, 16S-23S spacer region and a part of the 23S rRNA gene and the rp gene region of samples were deposited in Genbank as Banana wilt associated phytoplasma (BWAP) (Davis et al., 2012). According to these authors, during 2009 and 2010 surveys, positive identifications of phytoplasmas belonging to 16SrII, 16SrVIII, groups, the BWAP as well as an undetermined phytoplasma were obtained from samples of plants having leaf death, internal pockets and discontinuous streaking but not from plants with leaf yellowing alone. Phylogenetic analyses of the 16S rRNA gene showed that the phytoplasma from banana samples, clusters most closely with phytoplasmas associated with lethal yellowing type diseases of coconut in Papua New Guinea and other countries but do form a distinct lineage from all other phytoplasma groups.

### Epidemiology: Communalities and Differences

Epidemiological patterns are the result of many interacting factors, including populations of the causal organisms, host range, environmental conditions and management practices applied during disease outbreaks. Yet, bacterial diseases affecting banana and enset share many communalities on epidemiology that consequently drive a common set of management options.

*Xanthomonas* and *Ralstonia* spp. bacteria enter host plants via wounds that expose internal tissues. Such wounds may be either artificial or naturally appearing during plant development. The abscission of male flowers creates a moist site with open xylem vessels that can be inoculated by bees or other flying insects that carry the pathogen from diseased plants that are oozing bacteria on infected inflorescences (Buddenhagen and Kelman, 1964). Management practices using garden tools such as machetes may also create entry sites for bacteria (Ocimati et al., 2013b). In addition, nematodes may cause wounds enabling root entry for both *Xanthomonas* and *R. solanacearum* (Denny, 2006; Shehabu et al., 2010).

Insect-driven epidemics, for Xanthomonas wilt, Bugtok and blood disease often develop rapidly due to an abundant presence of insect vectors and the speed with which susceptible ABB or BBB cultvars (such as ‘Saba’ in the Philippines, ‘Pisang Kepok’ in Indonesia, ‘Pisang Awak’ and ‘Bluggoe’ in East and Central Africa) become infected/infectious (Blomme et al., 2005; Ploetz et al., 2015). Although Bugtok in the Philippines and Blood disease in Indonesia are caused by two different pathogens, *R. solanacearum* and *R. syzygii* subsp. *celebesensis* they have similarities in epidemiology. For example, both pathogens severely infect the same variety (called ‘Saba’ in the Philippines and ‘Pisang Kepok’ in Indonesia), a variety with balbisiana constitution (*Musa* BBB type). These balbisiana-derived varieties have a wide opening of the bracts thus exposing the fresh male flowers, and thus attracting a large number of insects to the inflorescence. It is a common observation that the male buds of this BBB variety attract more insects (e.g., flies, bees, and wasps), far more than the acuminata varieties (AAA), such as ‘Lakatan’ and ‘Latundan’ in the Philippines and ‘Pisang Berangan’ in Indonesia. An analysis of sugar contents of male flower nectar indicated that cultivars with balbisiana genomes tend to be sweeter, with more simple sugars compared to the other varieties (Dimyati et al., 2001). This result was not surprising, as balbisiana male flowers are an important vegetable in Asia because they are perceived as “sweeter” compared to the acuminatas, which are bitter (Molina, 2006). As a result, cultivars like Cavendish (AAA) and Lakatan (AAA), are not seriously affected by Bugtok under small-scale farmer conditions because insects do not prefer to feed on these varieties (Molina, 2006). More recent findings in Xanthomonas wilt affected zones revealed that though insect populations play an important role in disease spread, the observed high susceptibility of ABB or BBB cultvars is attributed to their non-persistent male and neutar flowers and bracts (Addis et al., 2004; Shimelash et al., 2008; Ocimati et al., 2013a; Rutikanga et al., 2016b). These male and neutar flowers/bracts leave behind fresh open wounds that act as entry points for the pathogen(s) on the body parts of visiting and foraging insects

Contaminated farm machinery, garden tools and machetes used for pruning and de-suckering, and infected fruit and rhizomes (used as planting material) are also effective vehicles of dissemination (Ploetz et al., 2015). Contaminated water reservoirs (for irrigation purposes) are extremely effective to disseminate *R. solanacearum* and are major constraints to control Moko in Latin America. For instance, in Colombia, in spite of a rigid Moko control program, over 20,000 cases of Moko were recorded during 2013 in Cavendish plantations in Santa Marta. The pathogen was mainly disseminated by water, either during flooding, via river water and/or drainage channels. In Mexico, flooding (and contaminated planting materials) allowed the introduction of Moko to disease-free areas (Fucikovsky and Santos, 1993). Currently there is no report or evidence of Xcm spread through soil or by water. Soil-related dispersions play a significant role for *Ralstonia* and *Dickeya*, but it is of limited importance for *Xcm* bacteria (Biruma et al., 2007). *R. solanacearum* has been reported to survive in agricultural soil up to 1 year even after eliminating host plants using herbicide (van Elsas et al., 2005). For example, BDB can survive in soil at least for a year in infested plant residues and infect the banana host roots (Gäumann, 1921). Infected soil, vehicles and tools move the blood disease pathogen within plantations and planting material and fruits are capable of spread at long distances (Buddenhagen, 2009). In contrast, the survival period of *Xcm* bacteria is limited, ranging from 9 to 35 days in plant debris or soil (Mwebaze et al., 2006; Welde-Michael et al., 2008) and saprophytic survival outside the host is limited, due to relatively slow multiplication rates, compared with *Ralstonia* or *Pseudomonas* (Biruma et al., 2007).

Latently infected planting materials are known to promote long-distance dispersal of bacterial wilt pathogens (Eden-Green, 2004; Molina, 2006). For example, the dispersion of Moko from Central America to the Philippines has been attributed to infected suckers (Rillo, 1979, 1981; Buddenhagen, 1986). In Indonesia, the movement of blood disease can also be traced with movements of planting materials and infected plant parts especially the balbisianas (ABB and BBB) since these are important cooking bananas and are used in socio-cultural events.

Nakato et al. (2013a) assessed the risk of spreading *Xcm* through asymptomatic mature bunches by traders. Samples of banana fingers and rachis were collected from markets within Kampala, Uganda and at border points of Uganda with the Democratic Republic of Congo, Kenya, Tanzania and Rwanda. The study demonstrated that *Xcm* is transported in traded bunches/fruits over long distances and across borders. For example, up to 53% of sampled bunches at Kampala markets contained Xcm, while up to 62% of assessed bunches tested positive at international borders.

Hence, quarantine and prevented movement of plant parts from infected to clean areas is imperative.

Strain-specific dispersion ability has not been studied for *Xcm* bacteria, although the two major sub-lineages identified by Wasukira et al. (2012) may prove to exhibit variable characteristics. By contrast, different strains of *R. solanacearum* can show markedly different speeds of transmission and severity of disease expression. For instance, B strains (see the section on causal agents of bacterial wilt diseases) are mainly soil-borne and transmitted by root-to-root contact and farm management practices such as pruning. Insects may transmit B strains, but this is, however, rare, as plants infected by B strains exude relatively little bacterial ooze. By contrast, SFR and A strains are readily insect-transmitted (Buddenhagen and Kelman, 1964). Trigona bees, wasps, and other insects have been reported to disseminate the SFR and, to a lesser extent, B strains (Stover, 1972; Buddenhagen, 1994; Jones, 2000; Ploetz et al., 2015).

Generalist insects and stingless bees, such as *Trigona* spp., feed on the nectar-like sap of banana plants, which exudes from fresh cushions where male flowers have fallen from their point of attachment. On blood disease infected plants, bacteria-filled droplets begin to ooze from such cushions about 15–25 days after infection. Although insects frequent both male and female flowers, these fresh cushions are the only surfaces containing open xylem vessels and nectar-like sap. The infection court (i.e., site in or on a host plant where infection can occur) is therefore not the flowers themselves, and only rarely the bract scars (Buddenhagen, 2009).

Dispersion by insects of the BDB (*R. syzygii* subsp. *celebesensis*) has been shown to occur at over 25 km per annum in some areas of Indonesia on cooking and dessert bananas (Eden-Green and Seal, 1993). Gäumann (1921) demonstrated blood disease transmission via the inflorescence. Mairawita et al. (2012) reported that the flying insect, *Trigona minangkabau*, was often infested with the blood disease pathogen in Sumatra. In Sulawesi, various large wasps, *Oncopsia* spp., *Trigona* bees and flies have been observed in contact with ooze discharging from peduncles and male buds of blood bacterial wilt-affected plants. Insects were also seen feeding on fresh cushions.

Tinzaara et al. (2006a,b) reported the vector potential of insects (e.g., stingless bees, honey bees, fruit flies and grass flies) in transmitting Xanthomonas wilt inoculum from male buds of infected plants to those of healthy plants. A reduced level of insect vector transmission of Xanthomonas wilt has been reported in Ethiopia, in North Kivu, eastern DR Congo and Rwanda at altitudes above 1,700 m above sea level (masl) (Addis et al., 2004; Rutikanga et al., 2015). It is postulated that the lower temperatures are not favorable for insect vectors.

The occurrence of isolated cases of Xanthomonas wilt in remote places in various districts across Uganda far from the originally identified disease sites suggested the involvement of long distance vectors in the transmission. Buregyeya et al. (2008, 2014) reported that birds [especially the eastern gray plantain eater (*Criniferzonurus*), double toothed barbet (*Lybins bidentatus*), sunbird (*Nectariniidae spp.*), and village weaverbird (*Ploceidae spp.*)] and bats (especially *Aidulon helvum, Epomophorus labiatus*, and *Epomaps franquet*) visit fruits and male flowers of banana and potentially pick up *Xcm*. The bacterium can survive up to 3 days on facial hairs of bats and up to 5 days on birds, making these animals potential long distance transmitters for the disease (Buregyeya et al., 2008, 2014). Since these animals mostly forage on male flowers, the early removal of male buds (as is recommended to prevent insect vector transmission) from bananas would limit disease spread.

Agricultural practices such as the use of cableways to transport bunches and tools from the plantations to packinghouses may also be important for bacterial dispersion. Munar-Vivas et al. (2010) used field-integrated information in geographical information system (GIS)-based maps to evaluate the presence of Moko in the Urabá region of Colombia, during three different time periods. They showed that 76% of Moko detected during the three time periods was associated with cableways used for transporting fruits and field consumables.

Disease progression is largely dependent on host susceptibility, environmental factors, existence of contaminated water sources and management practices. Incubation periods may vary depending on the maturity of the infected plant, method of inoculation, route of infection, and environmental conditions.

In the case of Xanthomonas wilt, higher incidence levels are often observed in the rainy season compared to the dry season (Shimwela et al., 2016), which may be due to higher water levels in plant tissue, favoring bacteria development. Caution must thus be taken when cutting diseased plants in the rainy season as higher inoculum levels may cause increased disease transmission rates when tool sterilization is not carried out.

*Dickeya paradisiaca* infects the plants through open entries and wounds produced during sanitation of senescent leaves attacked by black Sigatoka and pruning of suckers (Rivera, 1978; Thwaites et al., 2000). Cultivars of plantain (AAB) and cooking bananas (ABB) are more susceptible to pseudostem wet rot than Cavendish cultivars. Severe epidemic outbreaks are commonly observed after long periods with water deficit during the hot dry seasons in Central America. These conditions associated to poor sanitations practices enable severe symptom development including plant toppling.

According Davis and Ruabete (2010), all Papua New Guinea phytoplasma records so far known are from herbaceous dicotyledonous hosts. An important exception is the 16SrIV (coconut lethal yellowing) group phytoplasma, associated with severe disease in coconut palms in MaP suggesting that banana should be investigated as a possible alternative host in PNG’s coconut epidemics (Davis et al., 2012). Based on PCR detection, cloning and sequencing, the BWAP was also found in banana plants from different places of Papua New Guinea with abundant coconuts and showing no signs of phytoplasma like disease. The apparent lack of phytoplasma transfer between host species were explained by so far unidentified differences between BWAP or because difference in feeding behavior of vectors present (Davis et al., 2012). Further investigations into the phytoplasma disease status of monocotyledonous crops and weeds as well as studies to determine the insect vectors are essential to develop management strategies in banana and possibly coconut crops.

### Disease Management: Communalities and Differences

Good progress has been made regarding disease control/ management using innovative/improved cultural/agronomic practices in large, medium and small-scale farm settings. Integration of cultural practices with sensitive and specific diagnostic tools, transgenic approaches and conventional breeding may offer a more sustainable and environmentally friendly approach to control bacterial diseases.

Here we describe control methods that are elementary for all bacterial diseases of *Musa* spp. and more specific control strategies pertaining to specific pathogens. Control methods that are currently still under development will also be discussed. In general, key factors for management success are systematic and disciplined adoption and execution of monitoring and eradication of infected plants.

A first critical step in plant disease management is diagnosis. Disease recognition in banana plants affected by bacteria is achieved by plant-by-plant inspection of the plantation at regular intervals. Although the appearance of infected plants may differ depending on the cultivar, mode of disease transmission, plant growth stage and environmental conditions, available data on the average incubation period suggest that inspections need to be done at weekly intervals (Lehmann-Danzinger, 1987). The earliest appearance of Moko symptoms is 2 weeks after infection (Lehmann-Danzinger, 1987), while Xanthomonas wilt symptoms are typically evident within 2 weeks to 1 month depending on the entry point of the pathogen and age of the plants (Ssekiwoko et al., 2006a; Welde-Michael et al., 2008; Addis et al., 2010; Ocimati et al., 2013a). Although it has been advised to monitor fields/plots at weekly intervals for disease symptoms, depending on local circumstances, cultivar and strain present in the region, longer monitoring periods might be implemented.

#### Cultural Control

In regions, villages or farms where bacterial diseases are not present, the first line of defense is to avoid introducing them, i.e., through exclusion. Use of clean planting material and good sanitation procedures need to be always coupled to quarantine methods. For example, sanitation programs carried out in Cuba through systematic use of ELISA-indexed tissue culture plants were very successful in eliminating necrotic rhizome rot in Cavendish plantations (Pérez-Vicente, 2003). As *D. paradisiaca* grows well in meristem-growing media, infected plant material that was not detected during diagnostic indexing, can be readily discarded during the multiplication process leading to its total disappearance from the system after six multiplication steps (Hernández et al., 1994).

Where a disease is already endemic, control options should focus on a systematic area-wide approach, with the adoption of a combination of activities such as: limitation of access of animals, workers/laborers and equipment from and to the infected fields, regular disinfection of farm tools, implementing disinfection points in frequent access points, killing and removing diseased and neighbouring plants/mats, building channels around the infected plants to limit the movement of superficial water with bacterial inoculum, elimination of secondary host plants, removal of male flowers (de-budding) and early bagging of fruit clusters.

The male inflorescence part is the primary infection site for insect vectors and no infection occurs when male buds are removed just after the formation of the last fruit hand, i.e., before the first cushion of male flowers is exposed. The practice of de-budding by means of a forked wooden stick just after the formation of the last hand is an effective control measure for all bacterial wilts of *Musa* spp., incidentally also resulting in larger/bigger and more evenly filled fruits (Stover, 1972, cited in Soguilon et al., 1995; Sequeira, 1998; Blomme et al., 2005). A forked stick is used to avoid cross-infections associated with farm tools such as knives, machetes and sickles. In the Philippines, the effective management of Bugtok disease in commercial and backyard plantings of Saba (BBB) was demonstrated in extensive farmer field trials where early de-budding and fruit bagging with plastic bags were implemented (Molina, 1996). Molina also showed that the sole application of early male bud removal was sufficient to effectively reduce Bugtok infections. In addition, Opina et al. (1999) confirmed the effectiveness of early de-budding in managing the Bugtok disease in out-scaling trials in the Philippines. This practice is now advocated as a standard management practice in BBB Saba production systems. All the farmer field and out-scaling trials also provided empirical evidence that the inoculation route is generally through the male inflorescence part (and not the female part), and that transmission is primarily by insects (Molina, 2006).

A de-budding field trial, using the BBB cultivar ‘Pisang Kepok,’ was carried out in Indonesia in 1998–1999 by Catur Hermanto and Lilik Setyubodi of the Research Institute for Fruits. The results showed that early de-budding prevented insect vector transmission of the BDB in line with results obtained from de-budding trials carried out on Bugtok disease in the Philippines (Soguilon et al., 1995; Molina, 1996, 1999). Similar reports have been given for Xanthomonas wilt (Blomme et al., 2005). The role of the male inflorescence part in insect vector transmission is supported by observations in Indonesian that a ‘Pisang Kepok’-type cultivar which does not produce a male inflorescence part, is significantly less affected by banana blood disease (Molina, 1999).

Although de-budding is standard practice for commercial plantations (e.g., for Moko in Latin America or Bugtok in the Philippines), but remains inconsistently applied by farming communities suffering from Xanthomonas wilt in East and Central Africa (Kagezi et al., 2006; Mwangi and Nakato, 2007). Kagezi et al. (2006) and Muhangi et al. (2006) stated that the majority of farmers remove the male buds only sporadically and often too late to be fully effective in preventing insect vector-mediated transmissions of *Xcm.*

Other cultural management strategies aimed at the reduction of insect vectors include bagging the inflorescence shortly after emergence with a polyethylene bag, muslin cloth, or a fine nylon mesh bag. Bags can be removed after all the fruits have set if followed by removal of the male inflorescence. This bagging practice is common in commercial plantations in, e.g., Latin America, as it reduces not only bacterial wilt diseases, but also a whole range of insect-mediated fruit damage. It should be combined with mat and field sanitation, and removal of old, dead leaves. Injecting the male inflorescence with insecticide, as practiced by commercial plantation to control thrips, was not as effective against Bugtok disease as bagging (Soguilon et al., 1995).

Cleaning of garden/field tools during routine plantation and sanitation practices, in fields with *Xcm* or *Ralstonia*, can be done using a 20% solution of household bleach (sodium hypochlorite, NaOCl, 3.5%). Some ammonia-based disinfectants have proven to be effective in eradicating bacteria on farm tools, with the advantage that they are not corrosive, bio-degradable and more stable than sodium hypochlorite (Pérez-Vicente and Martínez de la Parte, 2015). A study by Nakato et al. (2013b) investigated the antibiotic potential of botanicals for the elimination of *Xcm* bacteria, alongside ash, cow urine and ‘waragi’ (a 40% local alcohol brew). 200 g of plant parts were crushed in 500 ml of distilled water and left to stand for at least an hour to allow the plant content to drain into the solution. This study reported that extracts from *Allium sativum, Carica papaya, Capsicum annum, Solanum lycopersicum*, and *Persea americana* eliminated over 90% of *Xcm* populations compared with 100% for NaOCl (3.5%). Fernández et al. (2013) compared different strategies for *D. paradisiaca* management in plantain and concluded that continuous tool sanitation with NaOCl (3.5%) reduced the disease intensity up to 80%. Tool disinfection using a fire (by holding the tool in the fire until the blade is too hot to touch) is an alternative and has been advocated for *Xcm* elimination in east and central Africa.

Buffer distances of over a mile without Bluggoe bananas can significantly reduce spread of Moko disease, although infrequently distances exceeding five miles have been bridged (Buddenhagen and Elsasser, 1962).

Roguing is an essential element of any disease control strategy. However, in the case of bananas and plantains, the laborious nature of uprooting a mat and then disposing of the infected materials severely compromises the effectiveness of this technique. For example, it takes one person a full day to completely excavate two mats (Mwangi, 2007), redirecting resources away from other more lucrative activities.

The removal/destruction of the infected *Musa* debris/materials has been cited numerous times as a hindrance to the implementation of region-wide Xanthomonas wilt control programs in East and Central Africa (Blomme et al., 2014). Digging a pit to bury infected plant debris is cumbersome and burning the debris is perhaps even more demanding, considering the large amounts of fuel wood required. In Indonesia, however, farmers managed to effectively control banana blood disease by burning uprooted material (Setyobudi and Hermanto, 1999).

Biosecurity Australia (2008) summarized factors influencing *R. solanacearum* causing the Moko disease in decomposing banana waste as follows: “The pathogen, in banana waste, would not be competitive because of its attenuated state after importation, relatively slow growth rate, lack of nutritional versatility and inability to cope with the stresses of exposure to solar radiation, desiccation and moderately high temperatures where it is likely to be restricted to the vascular tissue of the waste in dry conditions. In compost, the heat generated by micro–organism metabolism will kill the *R. solanacearum* in hours. Under wet conditions that favor saprophytes, the competition from a diverse microbial community growing in banana waste is likely to include members that produce lytic enzymes and antibiotic substances harmful to *R. solanacearum*. Taking these factors into consideration, the survival of *R. solanacearum* in banana waste will be limited to a very short period of time.”

Compared with the other major bacterial wilts affecting bananas and plantains, Xanthomonas wilt is almost uniquely a problem for small-scale farmers. This elicits a need to tailor management options that are able to meet the specific constraints of resource-poor farming systems. Recent research findings indicate that *Xcm* bacteria do not colonize all lateral shoots (i.e., partial/incomplete systemicity occurs; Ssekiwoko et al., 2006a; Ocimati et al., 2013a, 2015; Blomme et al., 2014) and, even when they do, that this does not necessarily or often lead to symptom expression and disease (i.e., latent infections occur). This finding led to the use of a control method whereby only the visibly diseased plants within a mat are cut off at soil level. The underlying idea is that the continued removal of diseased plants in a field reduces the inoculum level and lowers disease incidence below the economic threshold. It is hoped that single diseased stem removal (SDSR), together with the use of clean garden tools and de-budding will be effective and widely adopted by small-scale farmers affected by *Xcm* in East and Central Africa (Blomme et al., 2014). Whether SDSR would also be suited to control epidemics caused by other bacterial wilt pathogens is currently unclear and needs further investigation. Variable degrees of systemicity for *R. solanacearum* strains have been reported by Denny (2006), suggesting that it may be worthwhile to assess the SDSR technique under resource-poor farmer condition in areas affected by *R. solanacearum*. The current control method for Moko in medium to large-scale plantations in Central and South America comprises the continuous and timely destruction (using herbicides) of all infected mats and those located in a 5–8 m buffer radius around infected mats, coupled to strict restrictions in access to the treated sites until no new cases are reported.

Fallowing is particularly challenging for small-scale *Musa* farmers, as complete mat uprooting is labor intensive and complete removal of all corm pieces is often impossible, leading to subsequent shoot emergence. Additionally, farmers should monitor for weeds that may promote survival of pathogenic strains of bacterial wilt. The number of years that a rotation crop must be grown depends on the level of infestation, rigorousness of corm uprooting, survival capacity of the pathogen in local soils, climate. At least a one-, but more often a 2- or 3-year rotation or fallowing is required to reduce *Ralstonia* population levels below the damage threshold (Denny, 2006). Sequeira (1962) reported for *Ralstonia* that fallows of 18 months lead to less than 1% disease incidence 12 months after replanting. On the other hand, fallow periods varying between 6 and 12 months depending on the intensity of management in the systems have been recommended following infestation by Xanthomonas wilt (Turyagyenda et al., 2008; Sivirihauma et al., 2013; Rutikanga et al., 2016a). Farmers are often challenged in adopting fallow or crop rotation practices due to constraints of land availability and pressures to produce either a subsistence crop or one with high cash value. This is particularly the case when the field has been affected by Moko, Bugtok or banana blood disease, due to the wider host range of *Ralstonia* bacteria (Belalcazar et al., 2004). The efficiency of a fallow period is compromised by the ability of the pathogen to survive in the absence of the primary host crop, either in the soil or on plant species that persist during the fallow period. Removal of alternative weed hosts is recommended (Romo et al., 2012). Crop rotation has proven effective in reducing bacterial wilt populations. In Costa Rica, heavily infected banana plantations have successfully been rotated with velvet bean (*Mucuna pruriens*) for one or two cycles to reduce the *R. solanacearum* populations in the soil.

Alternate hosts may act as reservoirs for infection, complicating the implementation of control strategies, such as fallowing or crop rotation (Aritua et al., 2008). Ssekiwoko et al. (2006a) tested the host status of 20 different plant species and found that *Xcm* was only able to infect monocot plants belonging to two families (Musaceae and Cannaceae).

For the medium to big commercial Cavendish plantations in the Philippines, plant to plant transmission of Moko disease mainly occurs through tools used in regular de-suckering and pruning activities. Hence, infection commences from the basal parts or pseudostem. Insect transmission is rare since fruit bagging and early de-budding are standard plantation practices. In addition, it is very rare under commercial plantation conditions to find an inflorescence infection as early detection (Moko scouting) is done weekly and suspected un-shot infected plants are immediately eliminated, the infected mat quarantined and treated. Early Moko scouting is mainly based on early symptoms of wilting and chlorosis on un-shot plants. There are hence as good as no sources of inoculum for insect transmission. Hence, the major preventive measure in Cavendish plantations is “tool disinfestation” before and after pruning or de-suckering.

Untill about 10 years ago, treatment of infected mats in large-scale plantations in the Philippines, consisted of chopping down aerial plant parts and treating the soil through an application of methyl-bromide. After its ban, the complete removal of infected mats was tested out through the injection of 2,4-D and glyphosate. However, this method was perceived as too cumbersome and especially too slow. In general, infected mats need to be detected at an early stage and should not be allowed to stand for longer than 24 h after scouting/detection to avoid spread. The burning of rice hulls is now common practice. First, all aerial plant parts are chopped off all the way to the mat’s base. This is followed by burning rice hull (at two stages, i.e., immediately after chopping, and 3–4 weeks later) and subjecting the affected area to quarantine measures. Rice hulls burn slowly and totally inhibit the survival of bacteria. This method completely kills the remaining corms as well as the bacteria in the soil. In addition, pruning tool sterilization is carried out by using disinfectants as, e.g., Dowicide (a.i., sodium salt o-phenylphenate).

On the other hand, early detection/eradication are not practiced in small-scale balbisiana farms. Moreover, small-scale farmers in the Philippines or Indonesia do not bag the fruits and they do not de-bud early enough (if ever) to prevent inoculation. This situation favors inflorescence infection. Movement of the bacteria from the fruit to the base of the plant is relatively slow for Bugtok disease. In many instances, when a Bugtok infected plant is harvested, and the stem is cut at the base, no signs of bacteria/symptoms can be found at the base. Although insect vector mediated banana blood disease symptom development looked “more aggressive” in field epidemics in Indonesia compared to Bugtok disease in the Philippines, no specific systemicity and speed of bacterial movement research has been carried out on banana blood disease.

When incidence levels in Bugtok infected fields (on small-scale balbisiana farms) rise and tool transmissions occur (although rare as tool use is uncommon), more and more leaf yellowing and wilting symptoms will be observed. At such an advanced stage of the epidemic, eradication of mats, soil-treatments or even long fallow periods should be advocated, due to the survival of the Moko/Bugtok pathogen in the soil or plant residues.

In commercial plantations, a strict and sustained preventive management approach is implemented, based on the capacity to quantify the risk in terms of economics (the cost of control versus the cost of potential loss) and an in-depth understanding of the epidemiology of the disease within the intensive mono-cropped production system. In contrast, in the case of small scale farmers, facing Bugtok or banana blood diseases, however, simple the control package might be (de-budding and tool sterilization), adoption is not straightforward. Many of the balbisiana cooking varieties are grown in small plantings in backyards, on unattended land or even as volunteer plantings. The role of these cooking bananas in people’s livelihood strategies might hence not always be very significant. There will hence always be a good number of diseased plants left in the farming landscape that continuously provide sources of inoculum. In addition, a community approach to disease control most often does not work in small-scale farm setting. Similarly, poor adoption levels of tool sterilization have also been observed in Xanthomonas wilt infected regions of east Africa (Shimwela et al., 2016).

Soil amendments, such as organic matter (e.g., compost, rice husk powder and bagasse), inorganic fertilizers, or other material like oyster shell powder may modify soil microbial communities and result in suppressing the size or activity of the *R. solanacearum* population. These soil amendments have, however, not been widely studied and are not generally applied (Schönfeld et al., 2003; Lemaga et al., 2005; Saddler, 2005; van Elsas et al., 2005). Arenas et al. (2004) reported that incorporation of marigold (*Tagetes patula*) at 1kg/m^2^ reduced *R. solanacearum* population in plantains by 85%.

Under laboratory conditions, the application of potassium, nitrogen and calcium was identified as part of an integrated control package to reduce *Xcm* disease incidence and lengthen incubation periods (Atim et al., 2013). However, and in contrast, Ochola et al. (2014) reported that increasing fertilizer amounts did not significantly reduce (*p* > 0.05) disease incidence, wilt severity index or plant mortality for artificially inoculated banana plantlets in pot experiments.

#### Chemical Control

For many years the usual method of Moko control was to dig out the infected mat and apply methyl bromide. The negative impact of methyl bromide on the ozone layer, however, led to the ban of this substance in many countries. Dazomet (Basamid^®^granular 97%) provides an alternative solution as a soil sterilizer with good control of Moko/Bugtok disease. The methodology is similar to that for methyl bromide, the main advantage being that dazomet does not leave any harmful residues in the soil (Cronshaw, 1998).

Formalin has reportedly been used to drench the soil around *Ralstonia* infected Cavendish banana plants in the Philippines, resulting in lower bacterial counts (Pava et al., 2003). Also in the Philippines, farmers have said they sometimes use the insecticide and nematicide Furadan^®^(a.i. carbofuran 48%) on the mats of harvested, Bugtok-infected plants and report “no more Bugtok on new bunches harvested from the treated mat” (Pava et al., 2003).

A promising remedy for the control of Bugtok disease was the use of table salt, suggested by Jover, cited in Pava et al. (2003). Tested on the cultivars ‘Saba’ and ‘Cardaba’ 10 days before and/or 5 days after flowering, about 500 g of salt was poured into a hole bored into the supporting pseudostem. Water was then poured into the hole to dissolve the salt. Field trials gave promising results. The technology package (table salt at flowering in combination with bagging and de-budding) and disease-free planting materials were transferred to 972 farmers in 11 municipalities of Bukidnon (Philippines) during a 4-year project. Impact assessment of the project revealed a significant increase in surface area planted to the Bugtok-susceptible ‘Saba’ (Pava et al., 2003).

Chemical destruction using the injection of herbicides is an alternative that is 100-fold less labor intensive than roguing (Blomme et al., 2008). However, adoption by small-scale farmers affected by Xanthomonas wilt in east and central Africa has been minimal because herbicides are not always available in rural areas, the perceived high cost of herbicides, a reluctance to inject an already infected plant and reluctance to inject symptomatic plants, as physically attached asymptomatic plants might also die (Blomme et al., 2014). Herbicidal sprays have also been used in Central America in the fight against Moko disease (Lehmann-Danzinger, 1987). In Belize, Moko disease was effectively eradicated following systematic surveys of ABB type Bluggoe mats and smallholder dessert banana cultivars, coupled with glyphosate treatment of all infected mats and all adjacent mats within a 5 m radius around infected mats (Thwaites et al., 2000).

#### Biological Control

Biological control of *Xcm* through the use of antagonistic bacteria is still in an exploratory phase. In initial pot experiments in Ethiopia, four bacterial antagonists against Xanthomonas wilt were observed to reduce disease incidence by 56 to 75% (Abayneh, 2010). Initial laboratory-based studies on banana in Uganda have shown promising levels of *Xcm* suppression by some bacterial isolates namely *Burkholderia* spp., *Herbaspirillum* spp., and *Enterobacter* spp. isolated from banana tissues collected in different parts of Uganda (Were, 2016).

#### Cultivar Tolerance/Resistance, Conventional Breeding, and Genetic Engineering

Resistant cultivars represent an economical and environmentally benign disease control strategy (Boshou, 2005). Unfortunately, sources of resistance to bacterial wilt are often polygenic, making it difficult or impossible to transfer all the identified quantitative trait loci into desirable cultivars, in part due to linkage with undesirable traits (Boshou, 2005). A number of crops have been developed through conventional/traditional breeding that exhibit tolerance to *Ralstonia* wilts (i.e., satisfactory yield despite infection), including potato (French, 1994; Lemaga et al., 2005) eggplant (Gopalakrishnan et al., 2005), tobacco and tomato cultivars (Prior et al., 1994; Wang et al., 1998). Very little concerted effort has been made to develop *Musa* hybrids with such traits and extremely few cultivars or wild varieties have been found showing true resistance or tolerance (Soguilon et al., 1995; Sequeira, 1998; Ssekiwoko et al., 2006a).

The screening of East African Musa cultivars for reaction against Xcm in East Africa (Ssekiwoko et al., 2006a) yielded no resistant cultivars. Stover (1972) reported that “all varieties of commercial bananas and plantains are susceptible to Moko disease.” Bugtok disease is very common in backyards in the Philippines where ‘Saba’ and ‘Cardaba’ are planted. However, the following cultivars planted at the Davao National Crops Research and Development Center were also affected: ‘Mundo,’ ‘Turangkog,’ ‘Paa Dalaga,’ ‘Biguihan,’ ‘Inabaniko,’ and ‘Java’ (ABB/BBB genome); ‘Gubao,’ ‘Katsila,’ ‘Pelipita,’ ‘Madu-ranga,’ (ABB genome) and ‘Giant Kalapua’ (ABBB genome). This indicates that cultivars possessing the ‘B’ or *M. balbisiana* genome are susceptible to Bugtok (Soguilon et al., 1995).

None of a wide range of *Musa* cultivars (24), including commercial AAA cultivars (e.g., ‘Grande Naine,’ ‘Petit Naine,’ ‘Gros Michel,’ ‘Valery,’ etc…), plantains (AAB) and wild *Musa* (comprising *M. ornata, M. acuminata, M. balbisana, M. salaccensis*) appeared to be resistant to the BDB following stem inoculations (Baharuddin, 1994). This report is in line with Gäumann (1921) who did not find resistance in 100 cultivars tested against the pathogen. However, under normal farmer field conditions there is a significant difference in “susceptibility” among varieties. Sahlan and Nurhadi (1994) who carried out a field survey in 1994 in Indonesia reported that blood disease mainly affected *balbisiana* cooking varieties, namely ‘Pisang Kepok’ (‘Batu’), ‘Jimbluk,’ ‘Kapas,’ ‘Nangka,’ and ‘Kepok Besar’ and ‘Muli’ in West Sumatera, Lampung and West Java. This observation was confirmed by Hermanto and Setyobodi in 1998–1999 in a field survey in Sumatra island (Hermanto et al., 1998; Hermanto, 1999; Setyobudi and Hermanto, 1999 and Hermanto, 1999). These reports might indicate cultivar preferences of insect vectors, most likely linked to male flower nectar sweetness. In addition, Edison et al. (1996) and Setyobudi and Hermanto (1999) also reported that under field conditions, banana blood disease has been shown to affect primarily cooking bananas such as ‘Pisang Kepok,’ ‘P. Kapas,’ ‘P. Nangka,’ ‘P. Raja,’ and ‘P. Dewaka.’

In the case of Xanthomona wilt, the ABB type bananas ‘Pisang Awak’ and ‘Bluggoe’ with dehiscent bracts are especially vulnerable because of their attractiveness to flying insect vectors (Jones, 2000; Tripathi and Tripathi, 2009). Various reports (Denny, 2006; Buddenhagen, 2009) can be found in the literature of ‘escape’ cultivars, so-called because they possess physical barriers on the inflorescence preventing the entry of bacteria. They lack the male inflorescence or they have persistent male flowers and bracts preventing insect-mediated transmission. Other escape varieties have less attractive flowers or have abscission wounds/scars that fully dry or heal before the neutar/male flowers and bracts fall, and can thus not be penetrated by bacteria (Addis et al., 2004; Mwangi and Nakato, 2007; Tripathi and Tripathi, 2009). In the case of Bugtok, Pelipita (*Musa* ABB), which has persistent bracts, has been recommended as a replacement for other ABB type cooking bananas (Ploetz et al., 2015). The identification of cultivars with persistent male bracts/flowers or budless mutants (i.e., cultivars with only the female section of the terminal inflorescence) in populations of susceptible cultivars was postulated by Buddenhagen (2009) as a suitable solution for banana blood disease and by proxy for other bacterial wilts of *Musa* species, as the infection court is missing. In Indonesia, where banana blood disease is rampant on the cultivar ‘Pisang Kepok,’ other cultivars, such as ‘Pisang Susu’ (AAB genome) and ‘Pisang Mas’ (AA genome), are rarely diseased, while the disease has not been detected in ‘Pisang Raja’ (AAB genome), which has persistent bracts. In Sulawesi, a budless mutant of P. Kepok called ‘Pisang Puju’ is able to grow without expressing symptoms of banana blood disease. Efforts to propagate this mutant cultivar for widespread diffusion have thus far not been properly executed, despite good intentions and a brief collaboration with Chiquita in the early 1990s (Buddenhagen, 2009). Similarly, budless mutants of ‘Cardaba’ and ‘Bluggoe’ have been found. Despite the selection pressures of banana blood disease on local cultivars, such budless mutants remain rare in Sulawesi (Buddenhagen, 2009).

Similarly, in Central America, the clone ‘Pelipita’ (ABB genome) was distributed widely in the late 1960s as an SFR strain-resistant substitute for ‘Bluggoe’ after it was found to escape infection because of its persistent male bracts and flowers (Stover and Richardson, 1968). Bioversity International is currently evaluating, in different East African countries, *Musa* genotypes (AA, AAA, AAB, and ABB) with persistent bracts and flowers as potential alternatives to the *Xcm* susceptible ABB cultivar, ‘Pisang awak’/‘Kayinja.’ Unfortunately, ‘escape’ varieties can still become infected through other modes of transmission, such as contaminated garden tools.

The development of bacterial wilt-resistant plants through conventional breeding also suffers from the problems of long generation times, various levels of ploidy, sterility of most edible cultivars and limited genetic variability (Tripathi et al., 2004). However, following artificial inoculation of 31 diploid (AA) genotypes (21 natural germplasm and 10 hybrids) with the Moko pathogen in a greenhouse in Brazil by De Oliveira e Silva et al. (2000), the hybrids F2P2, 1741-01, 1319-01, and SH3362 and ‘Babi Yadefana,’ a cultivar from New Guinea, showed resistance to the Moko pathogen. This demonstrates the occurrence of genetic variability among diploid (AA) banana genotypes in their ability to express resistance to *R. solanacearum* strains causing Moko disease. Studies by Ssekiwoko et al. (2006a) have also identified *M. balbisiana*, a inedible wild relative of banana, as a potential source of resistance to Xanthomonas wilt. However, mechanisms of resistance toward Xcm in this wild banana still need to be elucidated. In contrast, *M. balbisiana* succumbed to BDB following stem inoculations (Baharuddin, 1994).

A collaborative research between the International Institute of Tropical Agriculture (IITA), the National Agricultural Research Organization (NARO)-Uganda, Academia Sinica (Taiwan) and the African Agricultural Technology Forum in Kenya has genetically engineered Xanthomonas wilt-resistant banana varieties using transgenes encoding for plant ferredoxin-like protein (*pflp*) and hypersensitive response assisting protein (*hrap*) isolated from sweet pepper (*C. annuum*). Both genes are defense genes which can intensify the hypersensitive response (Lin et al., 1997; Chen et al., 2000). Xanthomonas wilt resistant transgenic banana lines over-expressing these *hrap* and *pflp* genes have been successfully generated using agrobacterium-mediated transfer techniques and embryogenic cell suspensions and were evaluated under laboratory, screenhouse and controlled field conditions (Tripathi et al., 2010, 2014; Namukwaya et al., 2012). Transgenic lines that have proven resistant over three crop cycles in the field have been identified (Tripathi et al., 2014), but await a legal framework for release. This approach could potentially also be exploited for the control of other bacterial diseases, such as Moko/Bugtok and blood bacterial wilts.

## Conclusion

Bacterial diseases of banana continue to cause major losses in banana and plantain worldwide. While in some cases, such as Xanthomonas wilt, substantial efforts are already in place, research on Moko/Bugtok banana blood diseases and *Dickeya* is limited.

Acceptable management/control of bacterial disease in *Musa* is achievable by following strict, coordinated and integrated activities. These activities, when area wide performed in a systematic way and based on epidemiological parameters, may guarantee sustainable control. However, the current situations in Africa (for Xanthomonas wilt), Latin America and the Caribbean (for Moko and *Dickeya*) suggest that more efforts are needed at different levels. Growers, technicians and extension workers should be trained on disease recognition, epidemiology and management practices, with the support of plant protection experts.

In the current molecular era, it is promising that an integration of sensitive and specific diagnostic tools together with transgenic approaches, conventional breeding and screening for escape cultivars may offer environmentally friendly and less labor intensive options to control bacterial diseases.

## Author Contributions

All authors listed have made a substantial, direct and intellectual contribution to the work, and approved it for publication.

## Conflict of Interest Statement

The authors declare that the research was conducted in the absence of any commercial or financial relationships that could be construed as a potential conflict of interest.

## References

[B1] Abayneh T. B. (2010). *Evaluation of Biological Control Agents Against Bacterial wilt Pathogen* (*Xanthomonas campestris* pv. musacearum) *of Ensete (Ensete ventricosum)*. thesis submitted to the School of Graduate Studies of Addis Ababa University in partial fulfilment of the requirements for the degree of Master of Science in Applied Microbiology. Addis Ababa University George.

[B2] Abele S. Twine E. Legg C. (2007). *Food security in Rwanda. C3P Food Security Briefs No.5 Ibadan, Nigeria (IITA).* Availabe at: http://c3project.iita.org/

[B3] Addis T. Handoro F. Blomme G. (2004). Bacterial wilt (*Xanthomonas campestris* pv. musacearum) on Enset and banana in Ethiopia. *InfoMusa* 13 44–45.

[B4] Addis T. Turyagyenda L. F. Alemu T. Karamura E. Blomme G. (2010). Garden tool transmission of *Xanthomonas campestris* pv. musacearum on banana (*Musa* spp.) and enset in Ethiopia. *Acta Hort.* 879 367–372. 10.17660/ActaHortic.2010.879.39

[B5] Ailloud F. Lowe T. Cellier G. Roche D. Allen C. Prior P. (2015). Comparative genomic analysis of *Ralstonia solanacearum* reveals candidate genes for host specificity. *BMC Genomics* 16:270 10.1186/s12864-015-1474-8 PMC4396162 25888333

[B6] Albuquerque G. M. R. Santos L. A. Felix K. C. S. Rollemberg C. L. Silva A. M. F. Souza E. B. (2014). Moko disease causing strains of *Ralstonia solanacearum* from Brazil extend known diversity in paraphyletic phylotype II. *Phytopathology* 104 1175–1182. 10.1094/PHYTO-12-13-0334-R 24848276

[B7] Alvarez A. (2005). “Diversity and diagnosis of *Ralstonia solanacearum*,” in *Bacterial Wilt Disease and the Ralstonia solanacearum Species Complex* eds Allen C. Prior P. Hayward A. C. (St. Paul, MN: APS Press) 437–447.

[B8] Anonymous (2012). *Guide de Terrain sur le Contrôle et la Gestion du BXW (Wilt Bactérien du bananier) en Province du Nord et Sud Kivu, RDC.* North Kivu: Commission Provinciale de lutte contre les maladies du bananier au Nord Kivu. 27.

[B9] Arenas A. López D. Alvarez E. Llano G. Loke J. (2004). Efecto de prácticas agroecológicas sobre la población de *Ralstonia solanacearum* Smith causante de Moko de plátano. *Fitopatol. Colom.* 28 76–80.

[B10] Aritua V. Parkinson N. Thwaites R. Heeney J. V. Jones D. R. Tushemereirwe W. (2008). Characterization of the Xanthomonas sp. causing wilt of enset and banana and its proposed reclassification as a strain of *X. vasicola*. *Plant Pathol.* 57 170–177. 10.1111/j.1365-3059.2007.01687.x

[B11] Atim M. Beed F. Tusiime G. Tripathi L. van Asten P. (2013). High potassium, calcium and nitrogen application reduce susceptibility to banana Xanthomonas wilt caused by *Xanthomonas campestris* pv. musacearum. *Plant Dis.* 97 123–130. 10.1094/PDIS-07-12-0646-RE 30722270

[B12] Baharuddin B. (1994). Pathological, biochemical and serological characterization of the blood disease bacterium affecting banana and plantain (Musa spp.) in Indonesia. *Cuvill. Verlag Gottin.* 129.

[B13] Baharuddin B. Rudolph K. Niepold F. (1994). Production of monospecific antiserum against the blood disease bacterium affecting banana and plantain. *Phytopathology* 84 570–575. 10.1094/Phyto-84-570

[B14] Belalcazar S. C. Rosales F. E. Pocasangre L. E. (2004). “El Moko del banano y el plátano y el rol de las plantas hospederas en su epidemiología,” in *Proceedings of the XVI International ACORBAT Meeting. September 26-October 1* eds Orozco-Santos M. Orozco-Romero J. Robles-Gonzalez M. Velazquez-Monreal J. Medina-Urrutia V. Hernandez-Bautista J. A. (Oaxaca: Artturi) 16–35.

[B15] Biosecurity Australia (2008). *Final Import Risk Analysis Report for the Importation of Cavendish Bananas from the Philippines, Part C.* Canberra: Biosecurity Australia.

[B16] Biruma M. Pillay M. Tripathi L. Blomme G. Abele S. Mwangi M. (2007). Banana *Xanthomonas wilt*: a review of the disease, management strategies and future research directions. *Afr. J. Biotechnol.* 6 953–962. 10.4314/ajb.v6i8.56989

[B17] Blomme G. Jacobsen K. Ocimati W. Beed F. Ntamwira J. Sivirihauma C. (2014). Fine-tuning banana *Xanthomonas wilt* control options over the past decade in East and Central Africa. *Eur. J. Plant Pathol.* 139 265–281. 10.1007/s10658-014-0402-0

[B18] Blomme G. Mukasa H. Ssekiwoko F. Eden-Green S. (2005). On-farm assessment of banana bacterial wilt control options. *Afr. Crop Sci. Conf. Proc.* 7 317–320.

[B19] Blomme G. Turyagyenda L. F. Mukasa H. Eden-Green S. (2008). The effectiveness of different herbicides in the destruction of banana Xanthomonas wilt infected plants. Special Issue. Research Advances in Banana and Enset in Eastern Africa. *Afr. Crop Sci. J.* 16 103–110.

[B20] Boshou L. (2005). “A broad review and perspective on breeding for resistance to bacterial wilt,” in *Bacterial Wilt Disease and the Ralstonia solanacearum Species Complex* eds Allen C. Prior P. Hayward A. C. (St. Paul, MN: APS Press) 225–238.

[B21] Buddenhagen I. W. (1961). Bacterial wilt of bananas: history and known distribution. *Trop. Agric. (Trinidad)* 38 107–121.

[B22] Buddenhagen I. W. (1986). “Bacterial wilt revisited,” in *Bacterial wilt Disease in Asia and the South Pacific. Proceedings of an international workshop held at PCARRD, Los Baños, Philippines, 8 to 10 October 1985. ACIAR proceedings no. 13* ed. Persley G. J. (Canberra: Australian Centre for International Agricultural Research) 126–143.

[B23] Buddenhagen I. W. (1994). “Banana diseases caused by bacteria,” in *Compendium of Tropical Fruit Diseases* eds Ploetz R. C. Zentmyer G. A. Nishijima W. T. Rohrbach K. G. Ohr H. D. (St. Paul, Minnesota: APS Press) 15–17.

[B24] Buddenhagen I. W. (2009). Blood bacterial wilt of banana: history, field biology and solution. *Acta Hort.* 828 57–68. 10.17660/ActaHortic.2009.828.4

[B25] Buddenhagen I. W. Elsasser T. A. (1962). An insect-spread bacterial wilt epiphytic of Bluggoe banana. *Nature* 194 164–165. 10.1038/194164a0

[B26] Buddenhagen I. W. Kelman A. (1964). Biological and physiological aspects of bacterial wilt caused by *Pseudomonas solanacearum*. *Ann. Rev. Phytopathol.* 2 203–230. 10.1146/annurev.py.02.090164.001223

[B27] Buddenhagen I. W. Sequeira L. Kelman A. (1962). Designation of races in *Pseudomonas solanacearum*. *Phytopathology* 52:726.

[B28] Buregyeya H. Kubiriba J. Tusiime G. Kityo R. Ssekiwoko F. Tushemerierwe W. K. (2014). Role of birds and bats in long distance transmission of banana bacterial wilt in Uganda. *Int. J. Agric. Inno. Res.* 2 636–640.

[B29] Buregyeya H. G. Tusiime J. Kubiriba J. Tushmereirwe W. K. (2008). Evaluation of distant transmission of banana bacterial wilt in Uganda. *Paper presented at the conference on Banana and Plantain in Africa: Harnessing International Partnerships to Increase Research Impact, October* 5–9 2008 Mombasa.

[B30] CAB International (2014). *Invasive Species Compendium. Ralstonia solanacearum Race 2.* Available at: http://www.cabi.org/isc/datasheet/44999/aqb

[B31] Carter B. A. Reeder R. Mgenzi S. R. Kinyua Z. M. Mbaka J. N. Doyle K. (2010). Identification of *Xanthomonas vasicola* (formerly *X. campestris* pv. musacearum), causative organism of banana Xanthomonas wilt, in Tanzania, Kenya and Burundi. *Plant Pathol.* 59:403 10.1111/j.1365-3059.2009.02124.x

[B32] Castellani E. (1939). Su un marciume dell’ ensete. *L’Agric. Coloniale Firenze* 33 297–300.

[B33] Chen C. H. Lin H. J. Ger M. J. Chow D. Feng T. Y. (2000). The cloning and characterization of a hypersensitive response assisting protein that may be associated with the harpin- mediated hypersensitive response. *Plant Mol. Biol.* 43 429–438. 10.1023/A:1006448611432 11052195

[B34] Coenye T. Vandamme P. (2003). Simple sequence repeats and compositional bias in the bipartite *Ralstonia solanacearum* GM11000 genome. *BMC Genomics* 4:10 10.1186/1471-2164-4-10 PMC153513 12697060

[B35] Cronshaw D. K. (1998). “Basamid granular for the control of Moko disease,” in *Memorias XII Reunión Internacional de ACORBAT* ed. Guzmán Chaves J. A. San José 312–315.

[B36] da Silva A. R. Ferro J. A. Reinach F. C. Farah C. S. Furlan L. R. Quaggio R. B. (2002). Comparison of the genomes of two *Xanthomonas pathogens* with differing host specificities. *Nature* 417 459–463. 10.1038/417459a 12024217

[B37] Daniells J. W. (2011). Combating banana wilts: what do resistant cultivars have to offer? *Acta Hortic.* 897 403–411. 10.17660/ActaHortic.2011.897.56

[B38] Davis R. I. Kokoa P. Jones L. M. Mackie J. Constable E. Rodoni B. C. (2012). A new wilt disease of banana plants associated with phytoplasmas in Papua New Guinea (PNG). *Aust. Plant Dis. Notes* 7 91–97. 10.1007/s13314-012-0056-8

[B39] Davis R. I. Moore N. Y. Fegan M. (2001). “Blood disease and panama disease: two newly introduced and grave threats to banana production on the island of New Guinea,” in *Proceedings of the Papua New Guinea Food and Nutrition 2000 Conference* (Canberra: ACIAR) 26–30.

[B40] Davis R. I. Ruabete T. K. (2010). Records of plant pathogenic viruses and virus-like agents from twenty-two Pacific island countries/territories: a review and an update, Australas. *Plant Pathol.* 39 265–291. 10.1071/AP10047

[B41] De Oliveira e Silva S. de Mello Veras S. Gasparotti L. Pires de Mato A. Cordeiro L. Boher B. (2000). Evaluation of Musa spp. for resistance to Moko disease (Ralstonia solanacearum, race 2). *InfoMusa* 9 19–20.

[B42] Denny T. P. (2006). “Plant pathogenic ralstonia species,” in *Plant-Associated Bacteria* ed. Gnanamanickam S. S. (Dordrecht: Springer) 573–644. 10.1007/978-1-4020-4538-7_16

[B43] Denny T. P. Carney B. F. Schell M. A. (1990). Inactivation of multiple virulence genes reduces the ability of *Pseudomonas solanacearum* to cause wilt symptoms. *Mol. Plant-Microbe Interact.* 3 293–300. 10.1094/MPMI-3-293

[B44] Denny T. P. Hayward A. C. (2001). “Ralstonia,” in *Laboratory Guide for Identification of Plant Pathogenic Bacteria* 3rd Edn eds Schaad N. Jones J. B. Chun W. (St Paul, MN: APS Press) 165–189.

[B45] Dickey R. S. Victoria J. I. (1980). Taxonomy and emended description of strains of Erwinia isolated from *Musa paradisiaca* linnaeus. *Int. J. Syst. Bacteriol.* 30 129–134. 10.1099/00207713-30-1-129

[B46] Dimyati A. Djatnika Hermanto C. Nasir N. Hasyim A. (2001). “Current research activities on banana diseases and pests in Indonesia,” in *Proceedings of the 10th INIBAP-ASPNET Regional Advisory Committee meeting held at Bangkok, Thailand, 10-11 November 2000: Advancing Banana and Plantain R & D in Asia and the Pacific* Vol. 10 eds Molina A. B. Maghuyop V. N. Roa M. A. G. (Los Baños, Laguna: International Network for the Improvement of Banana and Plantain - Asia and the Pacific Network) 110–122.

[B47] Dita M. Garming H. Van den Bergh I. Staver C. Lescot T. (2013). Banana in latin america and the caribbean: current state. *Challeng. Perspect. Acta Hort.* 986 365–380. 10.17660/ActaHortic.2013.986.39

[B48] Dye D. W. (1969). A taxonomic study of the genus erwinia I, the amylovora group. *N. Z. J. Sci.* 12 81–97.

[B49] Dye D. W. Bradbury J. F. Goto M. Hayward A. C. Lelliot R. A. Schroth M. N. (1980). International standards for naming pathovars of phytopathogenic bacteria and a list of pathovar names and pathotype strains. *Rev. Plant Pathol.* 59 153–168.

[B50] Eden-Green S. J. (1994a). “Diversity of *Pseudomonas solanacearum* and related bacteria in southeast asia: new directions for Moko disease,” in *Bacterial wilt: The Disease and its Causative Agent, Pseudomonas solanacearum* eds Hayward A. C. Hartman G. L. (Wallingford: CAB International) 25–34.

[B51] Eden-Green S. J. (1994b). Banana Blood Disease. *INIBAP Musa Disease Fact Sheet No.* 3 Roma: Food And Agriculture Organization of The United Nation.

[B52] Eden-Green S. J. (2004). How can the advance of banana *Xanthomonas wilt* be halted? *InfoMusa* 13 38–41.

[B53] Eden-Green S. J. (2006). “Banana bacterial wilts: the global picture,” in *Proceeding of the Banana Xanthomonas wilt regional preparedness and strategy development workshop held, Kampala, Uganda: Developping a Regional Strategy to Address the Outbreak of Banana Xanthomonas wilt in East and Central Africa Kampala 14-18 February 2005* eds Karamura E. B. Osiru M. Blomme G. Lusty C. Picq C. (Montpellier: INIBAP) 12.

[B54] Eden-Green S. J. Seal S. E. (1993). “Bacterial diseases of banana and plantain in southeast asia,” in *Proceedings of an International Symposium on Genetic Improvement of Bananas for Resistance to Diseases and Pests: Breeding Bananas and Plantains for Resistance to Diseases and Pests, Montpellier, France, 7-9 September, 1992* eds Ganry J. (Montpellier: INIBAP) 115–121.

[B55] Edison H. S. Sutanto A. Hermanto C. Uji T. Razak N. (1996). *The Exploration of Musaceae in Maluku Island.* Hano: Research Institute for Fruits and the International Network for the Improvement of Banana and Plantain 63.

[B56] Elphinstone J. G. (2005). “The current bacterial wilt situation: a global view,” in *Bacterial wilt Disease and the Ralstonia solanacearum Species Complex* eds Allen C. Prior P. Hayward A. C. (St. Paul, MN: APS Press) 9–28.

[B57] Eyres N. Hammond N. Mackie A. (2005). *Moko Disease Ralstonia solanacearum.* Available at: www.agric.wa.gov.au

[B58] FAOSTAT (2014). *Banana and Plantain Surface and Production in 2013.* Available at: http://faostat.fao.org/site/339/default.aspx

[B59] Fegan M. (2005). “Bacterial wilt diseases of banana: evolution and ecology,” in *Bacterial wilt Disease and the Ralstonia solanacearum Species Complex* eds Allen C. Prior P. Hayward A. C. (St. Paul, MN: APS Press) 379–386.

[B60] Fegan M. Prior P. (2005). “How complex is the “Ralstonia solanacearum species complex,” in *Bacterial Wilt Disease and the Ralstonia solanacearum Species Complex* eds Allen C. Prior P. Hayward A. C. (St. Paul, MN: APS Press) 449–461.

[B61] Fegan M. Prior P. (2006). Diverse members of the *Ralstonia solanacearum* species complex cause bacterial wilts of banana. *Aust. Plant Pathol.* 35 93–101. 10.1186/s12864-016-2413-z

[B62] Fernández B. D. (1967). Pudrición acuosa del pseudotallo del plátano (*Musa paradisiaca*) causada por una especie de Erwinia. *Cenicafe* 18 39–46.

[B63] Fernández B. D. López D. G. (1970). Pudrición acuosa del pseudotallo del plátano (*Musa paradisiaca*) causada por Erwinia chysanthemi N. sp. *Cenicafe* 21 1–44.

[B64] Fernández J. Chavarría U. Brown D. Dita M. A. (2013). *Evaluación Preliminar de Tratamientos Para el Manejo de la Pudrición por Erwinia en Plátano en Rivas, Nicaragua*. *Resúmenes del II Congreso Latinoamericano y del Caribe de Plátanos y Bananos, Armenia, Colombia.* Maccarese: Bioversity international.

[B65] French E. R. (1986). “Interaction between strains of *Pseudomonas* solanacearum, its hosts and the environment,” in *Proceedings of the Bacterial Wilt Disease in Asia and the South Pacific. ACIAR Proceedings No. 13* eds Persley G. J. (Canberra: Australian Centre for International Agricultural Research) 99–104.

[B66] French E. R. (1994). “Strategies for integrated control of bacterial wilt of potatoes,” in *Bacterial wilt: The Disease and Its Causative Agent, Pseudomonas solanacearum* eds Hayward A. C. Hartman G. L. (Wallingford: CAB International) 199–207.

[B67] Fucikovsky L. Z. Santos M. O. (1993). “Advance of bacterial wilt in bananas in Mexico,” in *Proceedings of an International Conference Held on 28-31 October, 341-342* eds Hartman G. L. Hayward A. C. (Kaohsiung: IDEAS).

[B68] Gäumann E. (1921). Onderzoekingen over de bloedziekte der bananen op Celebes I. *Mededelingen van het*. *Instituut voor Plantenziekten* 50 1–47.

[B69] Genin S. Denny T. P. (2012). Pathogenomics of the *Ralstonia solanacearum* species complex. *Annu. rev. Phytopathol.* 50 67–89. 10.1146/annurev-phyto-081211-173000 22559068

[B70] Gillings M. R. Fahy P. (1994). “Genomic fingerprinting: towards a unified view of the *Pseudomonas solanacearum* species complex,” in *Bacterial wilt: the Disease and Its Causative Agent, Pseudomonas solanacearum* eds Hayward A. C. Hartman G. L. (Wallingford: CAB International) 95–112.

[B71] Gopalakrishnan T. R. Singh P. K. Sheela K. B. Shankar M. A. Kutty P. C. J. Peter K. V. (2005). “Development of bacterial wilt resistant varieties and basis of resistance in eggplant (Solanum melongena L.),” in *Bacterial wilt Disease and the Ralstonia solanacearum Species Complex* eds Allen C. Prior P. Hayward A. C. (St. Paul, MN: APS Press) 293–300.

[B72] Hayward A. C. (1964). Characteristics of *Pseudomonas solanacearum*. *J. Appl. Bacteriol.* 27 265–277. 10.1111/j.1365-2672.1964.tb04912.x

[B73] Hayward A. C. (1991). Biology and epidemiology of bacterial wilt caused by *Pseudomonas solanacearum*. *Annu. Rev. Phytopathol.* 29 65–87. 10.1146/annurev.py.29.090191.000433 18479193

[B74] Hayward A. C. (1994a). Systematics and phylogeny of *Pseudomonas* solanacearum. *Annu. Rev. Phytopathol.* 29 65–87. 10.1146/annurev.py.29.090191.000433 18479193

[B75] Hayward A. C. (1994b). “The hosts of *Pseudomonas solanacearum*,” in *Bacterial wilt: The Disease and its Causative Agent, Pseudomonas solanacearum* eds Hayward A. C. Hartman G. L. (Wallingford: CAB International) 9–24.

[B76] Heng S. B. (2012). *Blood Disease, Banana: Malaysia. ProMed 20120106.10021287.* Available at: http://www.promedmail.org/

[B77] Hermanto C. (1999). *Pengumpulan Isolat-Isolat Bakteri Petogenik Pada Tanaman Buah di Jawa Timur.* Laporan Perjalanan Dinas No. 268.P/BS/98. Belum di publikasi. 31 hal.

[B78] Hermanto C. Setyawati T. Santoso P. J. (1998). *Konfirmasi: Daerah Endemik baru Penyakit Layu Bakteri Pisang di Sumatera Barat*. *Disampaikan pada Seminar Sehari PFI Komca Sumbar, Riau dan Jambi, Padang* 1998.

[B79] Hernández R. Herrera L. Pichardo T. Alvarado Y. (1994). Diagnóstico de erwinia chrysanthemi Burk et al. en el proceso de micropropagación in vitro del plátano (Musa spp.). *Centro Agríc.* 21 62–67.

[B80] Hevesi M. Rivera N. Perez L. (1981). Medio selectivo para aislar y conservar a Erwinia chrysanthemi. Ciencia y Técnica en la Agricultura. *Ser. Prot. Plant.* 4 61–70.

[B81] Ilagan Y. A. Lavina W. A. Natural M. P. Raymundo A. K. (2003). Genetic homogeneity of the banana-infecting strains of *Ralstonia solanacearum* (Smith) Yabuuchi et al. in the Philippines. *Philipp. Agric. Sci.* 86 394–402.

[B82] Jones D. R. (2000). *Diseases of Banana, Abaca and Enset.* Oxfordshire: CABI.

[B83] Jones D. R. (2013). “Emerging banana diseases: new threats from old problems,” in *Proceedings of the XX Internationl Meeting ACORBAT* Fortaleza 79–90.

[B84] Kagezi G. H. Kangire A. Tushmereirwe W. Bagamba F. Kikulwe E. Muhangi J. (2006). Banana bacterial wilt incidence in Uganda. *Afr. Crop Sci. J.* 14 83–91.

[B85] Kalyebara R. Wood S. Abodi P. M. (2007). “Assessing the potential impact of selected technologies on the banana industry in Uganda,” in *An Economic Assessment of Banana Genetic Improvement and Innovation in the Lake Victoria Region of Uganda and Tanzania. IFPRI Research Report 155* eds Smale M. Tushemereirwe W. K. (Washington, DC: IFPRI) 141–156.

[B86] Karamura D. Mgenzi B. Karamura E. Sharrock S. (2004). Exploiting IK for the management and maintenance of Musa biodiversity on farm. *Afr. Crop Sci. J.* 12 71–78. 10.1186/s13002-016-0109-8

[B87] Karamura E. Frison E. Karamura D. A. Sharrock S. (1999). “Banana production systems in eastern and southern Africa,” in *Bananaa and Food Security* eds Picq C. Foure E. Frison E. A. (Wageningen: INIBAP) 401–412.

[B88] Karamura G. Smith J. Studholme D. Kubiriba J. Karamura E. (2015). Comparative pathogenicity studies of the *Xanthomonas vasicola* species on maize, sugarcane and banana. *Afr. J. Plant Sci.* 9 385–400. 10.5897/AJPS2015.1327

[B89] Kelman A. (1953). The bacterial wilt caused by *Pseudomonas solanacearum*. *Phytopathology* 55 304–309.

[B90] King E. O. Ward M. Ramy D. E. (1954). Two simple media for the demonstration of pyocianin and fluorescein. *J. Lab. Med.* 44 301–305. 13184240

[B91] Kogeethavani R. Sulastri N. J. Mazanah M. Rozeita L. Mohamad Roff M. N. (2013). *First Report of Blood Disease Bacterium on Banana in Malaysia.* Malaysia: Malaysian Agricultural Research and Development Institute.

[B92] Lee B.-M. Park Y.-J. Park D.-S. Kang H.-W. Kim J.-G. Song E.-S. (2005). The genome sequence of *Xanthomonas oryzae* pathovar oryzae KACC10331, the bacterial blight pathogen of rice. *Nucleic Acids Res.* 33 577–586. 10.1093/nar/gki206 15673718 PMC548351

[B93] Lehmann-Danzinger H. (1987). “The distribution of Moko disease in Central and South America and its control on plantains and bananas,” in *Proceedings of the CTA Seminar: Improving citrus and Banana Production in the Caribbean through Phyto-Sanitation, St Lucia* 130–152.

[B94] Lemaga B. Kakuhenzire R. Kassa B. Ewell P. T. Priou S. (2005). “Integrated control of potato bacterial wilt in eastern Africa: the experience of African Highlands Initiative,” in *Bacterial wilt Disease and the Ralstonia solanacearum Species Complex* eds Allen C. Prior P. Hayward A. C. (St. Paul, MN: APS Press) 145–157.

[B95] Lescot T. (2013). “World plantain and banana production systems,” in *Proceedings XX International Meeting ACORBAT. September 9 – 13, 2013* Fortaleza 26–34.

[B96] Lescot T. (2015). Genetic diversity of banana. Close-up. *Fruitrop* 231 98–102.

[B97] Lin H. J. Cheng H. Y. Chen C. H. Huang H. C. Feng T. Y. (1997). Plant amphipathic proteins delay the hypersensitive response caused by harpinPss and *Pseudomonas syringae* pv. syringae. *Physiol. Mol. Plant Pathol.* 51 367–376. 10.1006/pmpp.1997.0121

[B98] Llanos C. (1967). Una nueva enfermedad del plátano en el Valle del Cauca: la bacteriosis. *Agric. Trop.* 23 806–812.

[B99] Mairawita S. Habazar T. Hasyim A. Nasir N. (2012). “Trigona minangkabau potential as bacterial spreader agent of *Ralstonia solanacearum* phylotype IV cause blood disease on banana plants,” in *Proceedings of the Intetnational Proceedings of the Biology Life Science Singapore, July* 23–24 (Singapore: IACSIT Press) 109–116.

[B100] Mansfield J. Genin S. Magori S. Citovsky V. Sriariyanum M. Ronald P. (2012). Top 10 plant pathogenic bacteria in molecular plant pathology. *Mol. Plant Pathol.* 13 614–629. 10.1111/j.1364-3703.2012.00804.x 22672649 PMC6638704

[B101] Martini M. Lee I. M. Bottner K. D. Zhao Y. Botti S. Bertaccini A. (2007). Ribosomal protein gene-based phylogeny for finer differentiation and classification of phytoplasmas. *Int. J. Syst. Evol. Microbiol.* 57 2037–2051. 10.1099/ijs.0.65013-0 17766869

[B102] Mbaka J. N. Nakato V. G. Auma J. Odero B. (2009). Status of banana *Xanthomonas wilt* in Western Kenya and factors enhancing its spread. *Afr. Crop Sci. Conf. Proc.* 9 673–676.

[B103] Merchan-Vargas V. M. (2003). “Situación actual del Moko (Ralstonia solanacearum raza 2) en musáceas,” in *Manejo Convencional y Alternativo de Sigatoka negra, Nematodos y Otras Plagas Asociadas al Cultivo de Musáceas* eds Rivas G. Rosales F. (Guayaquil: MUSALAC/INIBAP) 181–182.

[B104] Mgenzi S. R. B. Muchunguzi D. Mutagwaba T. Mkondo F. Mohamed R. (2006). *An Outbreak of Banana Bacterial wilt in Muleba district, Kagera region, Tanzania.* Disease report, Bukoba: Maruku Agriculture Research and Development Institute Tanzania.

[B105] Minguez L. T. Natural M. P. Bayot R. (2011). Histological and morphological characterization of ‘Cardaba’ and ‘Cavendish’ roots of bananas (Musa x paradisciaca L. *infected with Ralstonia solanacearum, (Smith) Yabuuchi et al. ‘Race* 2’. *Philipp. J. Crop Sci.* 36 70.

[B106] Molina A. B. (1999). Fruit rot diseases of cooking banana in Southeast Asia. *Infomusa* 8 29–30.

[B107] Molina A. B. (2006). “Managing bacterial wilt/fruit rot disease of banana in Southeast Asia,” in *Proceedings of the Banana Xanthomonas wilt Regional Preparedness and Strategy Development Workshop Held in Kampala: Developping A Regional Strategy to Address the Outbreak of Banana Xanthomonas wilt in East and Central Africa* eds Karamura E. Osiru M. Blomme G. Lusty C. Picq C. (Montpellier: INIBAP) 26–31.

[B108] Molina G. C. (1996). Integrated management of “tibaglon”, a bacterial fruit rot disease of cooking bananas *Philippines. Phytopathology* 32 83–91.

[B109] Moreira L. M. Almeida N. F. Potnis N. Digiampietri L. A. Adi S. S. Bortolossi J. C. (2010). Novel insights into the genomic basis of citrus canker based on the genome sequences of two strains of *Xanthomonas fuscans* subsp. aurantifolii. *BMC Genomics* 11:238 10.1186/1471-2164-11-238 PMC2883993 20388224

[B110] Muhangi J. Nankinga C. Tushemereirwe W. K. Rutherford M. Ragama P. Nowakunda K. (2006). Impact of awareness campaigns for banana bacterial wilt control in Uganda. *Afr. Crop Sci. J.* 14 175–183.

[B111] Munar-Vivas O. Morales-Osorio J. G. Castañeda-Sánchez D. A. (2010). Use of field-integrated information in GIS-based maps to evaluate Moko disease (*Ralstonia solanacearum*) in banana growing farms in Colombia. *Crop Prot.* 29 936–941. 10.1016/j.cropro.2010.04.021

[B112] Mwangi M. (2007). *Removing Infected Banana Mats to Contain Xanthomonas wilt: Experiences in Uganda, Rwanda and the Democratic Republic of Congo. A brief prepared for the Crop Crisis Control Project. IITA-C3P* Kampala 13.

[B113] Mwangi M. Bandyopadhyay R. Tushemereirwe W. Ragama P. (2006). “Developing technologies to support replanting of banana to rehabilitate farms affected by *Xanthomonas wilt*,” in *Proceedings of the 4th International Bacterial wilt symposium, 17-20 July 2006* (York: Central Science Laboratory) 63.

[B114] Mwangi M. Nakato V. (2007). Key factors responsible for the banana *Xanthomonas wilt* pandemic on banana in East and Central Africa. *Acta Hort.* 828 395–404.

[B115] Mwebaze J. M. Tusime G. Tushmereirwe W. K. Kubiriba J. (2006). The survival of *Xanthomonas campestris* pv. musacearum in soil and plant debris. *Afr. Crop Sci. J.* 14 121–127.

[B116] Nakato G. V. Beed F. Ramathani I. Rwomushana I. Opio F. (2013a). Risk of banana *Xanthomonas wilt* spread through trade. *J. Crop Prot.* 2 151–161.

[B117] Nakato G. V. Ocimati W. Blomme G. Fiaboe K. K. M. Beed F. (2014). Comparative importance of infection routes for banana *Xanthomonas wilt* and implications on disease epidemiology and management. *Can. J. Plant Pathol.* 36 418–427. 10.1080/07060661.2014.959059

[B118] Nakato V. Ndugo V. Beed F. Ramathani I. Rwomushana I. Opio F. (2013b). Natural and synthetic disinfectants for farm tools contaminated with *Xanthomonas campestris* pv. musacearum. (in press). (ASARECA working series).

[B119] Namukwaya B. Tripathi L. Tripathi J. N. Arinaitwe G. Mukasa S. B. Tushemereirwe W. K. (2012). Transgenic banana expressing Pflp gene confers enhanced resistance to *Xanthomonas wilt* disease. *Transgenic Res.* 12 855–865. 10.1007/s11248-011-9574-y 22101927

[B120] Ndungo V. Bakelana K. Eden-Green S. Blomme G. (2004). An outbreak of banana *Xanthomonas wilt* (*Xanthomonas campestris* pv. musacearum) in the democratic republic of congo. *InfoMusa* 13 43–44. 10.1111/mpp.12578

[B121] Nurhadi M. dan Harlion R. (1994). Serangan bakteri dan cendawan pada tanaman pisang di propinsi dati I lampung [in indonesian]. *Info Hortik.* 2 37–40.

[B122] Ochola D. Ocimati W. Tinzaara W. Blomme G. Karamura E. (2014). Interactive effects of fertilizer and inoculum concentration on subsequent development of *Xanthomonas wilt* in banana. *Afr. J. Agr. Res.* 9 2727–2735. 10.5897/AJAR2014.8787

[B123] Ocimati W. Nakato G. V. Fiaboe K. K. M. Beed F. Blomme G. (2015). Incomplete systemic movement of *Xanthomonas campestris* pv. musacearum and the occurrence of latent infections in xanthomonas wilt-infected banana mats. *Plant Pathol.* 64 81–90. 10.1111/ppa.12233

[B124] Ocimati W. Ssekiwoko F. Buttibwa M. Karamura E. Tinzaara W. Eden-Green S. (2013c). “Systemicity and speed of movement of Xanthomonas campestris pv. musacearum in the banana plant after garden tool-mediated infection,” in *Banana Systems in the Humid Highlands of Sub-Saharan Africa: Enhancing Resilience and Productivity* eds Blomme G. Vanlauwe B. van Asten P. (Wallingford: CAB International) 101–108.

[B125] Ocimati W. Ssekiwoko F. Karamura E. Tinzaara W. Eden-Green S. Blomme G. (2013a). Systemicity of *Xanthomonas campestris* pv. musacearum and time to disease expression after inflorescence infection in East African highland and ‘Pisang Awak’ bananas in Uganda. *Plant Pathol.* 62 777–785. 10.1111/j.1365-3059.2012.02697.x

[B126] Ocimati W. Ssekiwoko F. Karamura E. B. Tinzaara W. Blomme G. (2013b). Does *Xanthomonas campestris* pv. musacearum colonize banana cord root tissue?. *Acta Hort.* 986 103–109. 10.17660/ActaHortic.2013.986.8

[B127] Ohobela M. Claflin L. E. (1987). The taxonomic position of *Xanthomonas campestris* pv. holcicola (Elliott 1930) Dye 1978 and *Xanthomonas campestris* pv. vasculorum (Cobb 1893) Dye 1978. *Phytopathology* 77 1766–1766.

[B128] Opina O. S. Molina A. B. Molina G. C. (1999). *Improved Management System for Saba Banana.* Los Banos: Project Report to PCARRD.

[B129] Ordosgoitti A. Santos P. R. Haddad G. O. (1974). La pudrición acuosa del pseudotallo del plátano y su presencia en tres regiones de Venezuela. *Rev. Agric. Venezuela* 24 247–258.

[B130] Palleroni N. J. Doudoroff M. (1971). Phenotypic characterization and deoxyribonucleic acid homologies of *Pseudomonas solanacearum*. *J. Bacteriol.* 107 690–696. 4937783 10.1128/jb.107.3.690-696.1971 PMC246989

[B131] Pava H. M. Franje N. F. Timario T. J. (2003). Banana pilot demonstration studies for bukidnon: table salt and early debudding to control ‘Bugtok’ disease of cooking banana cultivars ‘Saba’ and ‘Cardaba’philippines. *J. Crop Sci.* 28 31–43.

[B132] Pérez-Vicente L. (2003). “Manejo integrado de plagas y enfermedades en bananos y plátanos en Cuba,” in *Memorias del Taller Manejo Convencional y Alternativo de la Sigatoka Negra, Nematodos y Otras Plagas Asociadas al Cultivo de Musáceas en los Trópicos* eds Rivas G. Rosales F. E. Guayaquil 37–54.

[B133] Pérez-Vicente L. Martínez de la Parte E. (2015). “Efecto de un desinfectante de superficies a base de amonios cuaternarios sobre *Fusarium oxysporum* f. sp. cubense y Dickeya paradisiaca en bananos y plátanos,” in *Proceedings of the Resúmenes del III Congreso de la Red Latinoamericana de Musáceas (MUSALAC) organizado por MUSALAC* (Santa Catarina: Bioversity International y Embrapa. Turupá).

[B134] Pieretti I. Royer M. Barbe V. Carrere S. Koebnik R. Cociancich S. (2009). The complete genome sequence of *Xanthomonas albilineans* provides new insights into the reductive genome evolution of the xylem-limited Xanthomonadaceae. *BMC Genomics* 10:616 10.1186/1471-2164-10-616 PMC2810307 20017926

[B135] Ploetz R. C. Kema G. H. J. Ma L.-J. (2015). Impact of diseases on export and smallholder production of banana. *Ann. Rev. Phytopathol.* 53 13.1–13.20. 10.1146/annurev-phyto-080614-120305 26002290

[B136] Prior P. Ailloud F. Dalsing B. L. Remenant B. Sanchez B. Allen C. (2016). Genomic and proteomic evidence supporting the division of the plant pathogen *Ralstonia solanacearum* into three species. *BMC Genomics* 17:90 10.1186/s12864-016-2413-z PMC4736150 26830494

[B137] Prior P. Fegan M. (2005). Recent developments in the phylogeny and classification of *Ralstonia solanacearum*. *Acta Hort.* 695 127–136. 10.17660/ActaHortic.2005.695.14

[B138] Prior P. Grimault V. Schmit J. (1994). “Resistance to bacterial wilt (Pseudomonas solanacearum) in tomato: present status and prospects,” in *Bacterial Wilt: The Disease and Its Causative Agent, Pseudomonas solanacearum* eds Hayward A. C. Hartman G. L. (Wallingford: CAB International) 209–223.

[B139] Raymundo A. K. Aves-Ilagan Y. Denny T. P. (1998). “Analysis of genetic variation in a population of banana-infecting strains of *Ralstonia solanacearum*,” in *Bacterial Wilt Disease: Molecular and Ecological Aspects* eds Prior P. Allen C. Elphinstone J. (Berlin: Springer-Verlag) 56–60.

[B140] Raymundo A. K. Orlina M. E. Lavina W. A. Opina N. L. (2005). “Comparative genome plasticity of tomato and banana strains of *Ralstonia solanacearum* in the Philippines,” in *Bacterial wilt Disease and the Ralstonia solanacearum Species Complex* eds Allen C. Prior P. Hayward A. C. (St. Paul, MN: APS Press) 387–393.

[B141] Remenant B. de Cambiaire J. C. Cellier G. Jacobs J. M. Mangenot S. Barbe V. (2011). Ralstonia syzygii, the blood disease bacterium and some Asian *R. solanacearum* strains form a single genomic species despite divergent lifestyles. *PLoS ONE* 6:e24356 10.1371/journal.pone.0024356 PMC3169583 21931687

[B142] Rijks A. B. (1916). *Rapport Over een Onderzoek Naar de Pisangsterfte op de Saleiereilanden.* Rome: Food And Agriculture Organization of The United Nation.

[B143] Rillo A. R. (1979). Bacterial wilt of banana in the Philippines. *FAO Plant Protect. Bull.* 27 105–108.

[B144] Rillo A. R. (1981). Differences of *Pseudomonas solanacearum* EFS isolates in abacá and enset. *Philipp. Agric.* 64 329–334.

[B145] Rivera N. (1978). Estudio comparativo de dos nuevas enfermedades bacterianas en áreas plataneras de Cuba. *Agrotec. Cuba* 10 35–44.

[B146] Rivera N. Ezavin M. (1980). Necrosis del cormo del plátano causada por *Erwinia chrysanthemi*. *Ciencia Técnica Agric. Ser. Prot. Plantas* 18 59–69.

[B147] Rivera N. García A. Gil C. (1980). Caracterización patotípica de aislamientos de *Erwinia chrysanthemi* procedentes de plátano y maíz. *Ciencia Técn. Agric. Ser. Prot. Plantas* 3 47–60.

[B148] Roesmiyanto L. H. Hutagalung L. (1989). Blood disease (*P. celebesis*) on banana in Jeneponto – Sulawesi Selatan [abstract in English]. *Hortikultura* 27 39–41.

[B149] Romo J. P. Morales Osorio J. G. Yepes M. S. (2012). Identification of new hosts for *Ralstonia solanacearum* (Smith) race 2 from Colombia. *Rev. Prot. Veg.* 27 151–161.

[B150] Rorer J. B. (1911). A bacterial disease of bananas and plantains. *Phytopathology* 1 45–49.

[B151] Rutikanga A. Night G. Tusiime G. Ocimati W. Blomme G. (2015). “Spatial and temporal distribution of insect vectors of *Xanthomonas campestris* pv. musacearum and their activity across banana cultivars grown in Rwanda,” in *Proceedings of the 7th Congress on Plant Protection. Plant Protection Society of Serbia* eds Marčić D. Glavendekić M. Nicot P. (Belgrade: IOBC-EPRS) 139–153.

[B152] Rutikanga A. Sivirihauma C. Ocimati W. Night G. Murekezi C. Ndungo V. (2016a). Breaking the cycle of *Xanthomonas campestris* pv. musacearum in infected fields through the cultivation of annual crops and disease control in adjacent fields. *J. Phytopathol.* 164 659–670. 10.1111/jph.12489

[B153] Rutikanga A. Tusiime G. Night G. Ocimati W. Blomme G. (2016b). Variation in nectar volume and sugar content in male flowers of Musa cultivars grown in Rwanda and their non-effect on the numbers of visiting key diurnal insect vectors of banana *Xanthomonas wilt*. *Afr. J. Agric. Res.* 11 607–623. 10.5897/AJAR2015.10476

[B154] Saddler G. S. (2005). “Management of bacterial wilt disease,” in *Bacterial wilt Disease and the Ralstonia solanacearum Species Complex* eds Allen C. Prior P. Hayward A. C. (St. Paul, MN: APS Press) 121–132.

[B155] Safni I. Cleenwerck I. De Vos P. Fegan M. Sly L. Kappler U. (2014). Polyphasic taxonomic revision of the *Ralstonia solanacearum* species complex: proposal to emend the descriptions of *Ralstonia solanacearum* and *Ralstonia syzygii* and reclassify current *R. syzygii* strains as *Ralstonia syzygii* subsp. syzygii subsp. nov., *R. solanacearum* phylotype IV strains as *Ralstonia syzygii* subsp. indonesiensis subsp. nov., banana blood disease bacterium strains as *Ralstonia syzygii* subsp. celebesensis subsp. nov. and *R. solanacearum* phylotype I and III strains as *Ralstonia pseudosolanacearum* sp. nov. *Int. J. Syst. Evol. Microbiol.* 64 3087–3103. 10.1099/ijs.0.066712-0 24944341

[B156] Sahlan and Nurhadi. (1994). Inventarisasi penyakit pisang di sentra produksi Sumatera Barat, Jawa Barat dan Lampung. *Penel. Hort.* 6 36–43.

[B157] Samson R. Legendre J. B. Christen R. Achouak W. Gardan L. (2004). Transfer of *Pectobacterium chrysanthemi* (Brenner et al. 1973) Hauben et al. 1998 and *Brenneria paradisiaca* to the genus Dickeya gen. nov. as *Dickeya chrysanthemi* comb. nov. and *Dickeya paradisiaca* comb. nov. and delineation of four novel species: *Dickeya dadantii* sp. nov., *Dickeya dianthicola* sp. nov., *Dickeya dieffenbachiae* sp. nov. and *Dickeya zeae* sp. nov. *Int. J. Syst. Evol. Microbiol.* 55 1415–1427. 10.1099/ijs.0.02791-0 16014461

[B158] Schaad N. W. (1987). Problems with the pathovar concept. *Curr. Plant Sci. Biotechnol. Agric.* 4 783–785. 10.1007/978-94-009-3555-6_171

[B159] Schönfeld J. Gelsomino A. van Overbeek L. S. Gorissen A. Smalla K. van Elsas J. D. (2003). Effects of compost addition and simulated solarisation on the fate of *Ralstonia solanacearum* biovar 2 and indigenous bacteria in soil. *FEMS Microbiol. Ecol.* 43 63–74. 10.1111/j.1574-6941.2003.tb01046.x 19719697

[B160] Seal S. E. Elphinstone J. G. (1994). “Advances in identification and detection of *Pseudomonas solanacearum*,” in *Bacterial Wilt: The Disease and Its Causative Agent, Pseudomonas solanacearum* eds Hayward A. C. Hartman G. L. (Wallingford: CAB International) 37–57.

[B161] Seal S. E. Jackson L. A. Young J. P. W. Daniels M. J. (1993). Differentiation of *Pseudomonas solanacearum, Pseudomonas* syzygii, *Pseudomonas pickettii* and the blood disease bacterium by partial 16S rRNA sequencing: construction of oligonucleotide primers for sensitive detection by polymerase chain reaction. *Microbiololgy* 139 1587–1594. 10.1099/00221287-139-7-1587 8371118

[B162] Sequeira L. (1962). Control of bacterial wilt of banana by crop rotation and fallowing. *Trop. Agric. (Trinidad)* 38 211–217.

[B163] Sequeira L. (1998). “Bacterial wilt: the missing element in international banana improvement programs,” in *Bacterial Wilt Disease: Molecular and Ecological Aspects* eds Prior P. Allen C. Elphinstone J. (Berlin: Springer-Verlag) 6–14.

[B164] Sequeira L. Averre C. (1961). Distribution and pathogenicity of strains of *Pseudomonas solanacearum* from virgin soils in Costa Rica. *Plant Dis. Rep.* 45 435–440.

[B165] Setyobudi L. Hermanto C. (1999). Rehabilitation of cooking bananas from blood disease: Baseline status of distribution and infestation in Sumatera. *Paper presented on the RISBAP Meeting on September* 13-17 1999 at Phitsdanulok, Thailand, 6.

[B166] Shehabu M. Addis T. Mekonen S. De Waele D. Blomme G. (2010). Nematode infection predisposes banana to soil-borne *Xanthomonas campestris* pv. musacearum transmission. *Tree For. Sci. Biotechnol.* 4 63–64.

[B167] Shillingford C. A. (1974). Bacterial rhizome rot in Jamaica. *Plant Dis. Rep.* 58 214–218.

[B168] Shimelash D. Alemu T. Addis T. Turyagyenda F. L. Blomme G. (2008). Banana *Xanthomonas wilt* in Ethiopia: occurrence and insect vector transmission. *Afr. Crop Sci. J.* 16 75–87.

[B169] Shimwela M. M. Ploetz R. C. Beed F. D. Jones J. B. Blackburn J. K. Mkulila S. I. (2016). Banana *Xanthomonas wilt* continues to spread in Tanzania despite an intensive symptomatic plant removal campaign: an impending socio-economic and ecological disaster. *Food Secur.* 8 939–951. 10.1007/s12571-016-0609-3

[B170] Sivirihauma C. Rutikanga A. Murekezi C. Blomme G. Anuarite U. Ocimati W. (2013). “Effect of length of a fallow period after total uprooting of a *Xanthomonas wilt*-infected banana field on infection of newly established planting materials: case studies from Rwanda and North Kivu, Democratic Republic of Congo,” in *Banana Systems in the Humid Highlands of Sub-Saharan Africa: Enhancing Resilience and Productivity* eds Blomme G. Vanlauwe B. van Asten P. (Wallingford: CAB International) 125–130.

[B171] Smith E. F. (1896). A bacterial disease of the tomato, eggplant, and Irish potato (*Bacillus solanacearum* n. sp.). *Bull. Div. Veg. Physiol. Pathol. U.S Dep. Agric.* 12 1–28.

[B172] Soguilon C. E. Magnaye L. V. Natural M. P. (1995). Bugtok disease of banana. *Musa Disease Fact Sheet* 6 INIBAP, Montpellier France.

[B173] Ssekiwoko F. Taligola H. K. Tushmereirwe W. K. (2006a). *Xanthomonas campestris* pv. musacearum host range in Uganda. *Afric. Crop Sci. J.* 14 111–120.

[B174] Ssekiwoko F. Turyagyenda L. F. Mukasa H. Eden-Green S. Blomme G. (2006b). “Systemicity of *Xanthomonas campestris* pv. musacearum in flower-infected banana plants,” in *Proceedings of the XVII ACORBAT International Meeting: Banana: A Sustainable Business. Joinville* Santa Catarina.

[B175] Ssekiwoko F. Turyagyenda L. F. Mukasa H. Eden-Green S. Blomme G. (2010). Spread of *Xanthomonas campestris* pv. musacearum in banana (Musa spp.) plants following infection of the male inflorescence. *Acta Hortic.* 879 349–356. 10.17660/ActaHortic.2010.879.36

[B176] Ssekiwoko F. Tushemereirwe W. Batte M. Ragama P. E. Kumakech A. (2006c). Reaction of banana germplasm to inoculation with *Xanthomonas campestris* pv. musacearum. *Afric. Crop. Sci. J.* 14 151–155.

[B177] Stover R. H. (1972). *Banana, Plantain, and Abaca Diseases.* London: Commonwealth Agricultural Bureaux.

[B178] Stover R. H. Espinoza A. (1992). Blood disease of bananas in Sulawesi. *Fruits* 47 611–613.

[B179] Stover R. H. Richardson D. L. (1968). ‘Pelipita’, an ABB Bluggoe-type plantain resistant to bacterial and *Fusarial wilts*. *Plant Dis. Rep.* 52 901–903.

[B180] Stover R. H. Simmonds N. W. (1987). *Bananas* 3rd Edn London: Longmans.

[B181] Studholme D. J. Kemen E. MacLean D. Schornack S. Aritua V. Thwaites R. (2010). Genome-wide sequencing data reveals virulence factors implicated in banana *Xanthomonas wilt*. *FEMS Microbiol. Lett.* 310 182–192. 10.1111/j.1574-6968.2010.02065.x 20695894

[B182] Subijanto M. (1991). “Status of banana disease in Indonesia,” in *Proceedings of Technical Meeting on Diseases Affecting Banana and Plantain in Asia and the Pacific: Banana Diseases in Asia and the Pacific, Brisbane, Australia* eds Valmayor R. V. Umali B. E. Bejosano C. P. (Los Baños: INIBAP) 44–49.

[B183] Supriadi. (2005). “Present status of blood disease in indonesia,” in *Bacterial wilt Disease and the Ralstonia Species Complex* eds Allen C. Prior P. Hayward A. (Minnesota: APS Press) 395–404.

[B184] Taghavi M. Hayward C. Sly L. I. Fegan M. (1996). Analysis of the phylogenetic relationships of strains of *Burkholderia solanacearum, Pseudomonas* syzygii, and the blood disease bacterium of banana based on 16S rRNA gene sequences. *Int. J. Syst. Bacteriol.* 46 10–15. 10.1099/00207713-46-1-10 8573483

[B185] Teng S. K. Aziz N. A. A. Mustafa M. Laboh R. Ismail I. S. Sulaiman S. R. (2016). The occurrence of blood disease of banana in selangor, Malaysia. *Int. J. Agric. Biol.* 18 92–97. 10.17957/IJAB/15.0067

[B186] Thurston H. D. Galindo J. J. (1989). “Moko del banano y el plátano,” in *Enfermedades de Cultivos en el Trópico* (Turrialba: CATIE) 125–133.

[B187] Thwaites R. Eden-Green S. J. Black R. (2000). “Diseases caused by bacteria,” in *Diseases of Banana, Abacá and Enset* ed. Jones D. R. (Wallingford: CAB International) 213–239.

[B188] Thwaites R. Mansfield J. Eden-Green S. Seal S. (1999). RAPD and rep PCR-based fingerprinting of vascular bacterial pathogens of Musa spp. *Plant Pathol.* 48 121–128. 10.1046/j.1365-3059.1999.00321.x

[B189] Tinzaara W. Gold C. S. Ssekiwoko F. Tushemereirwe W. Bandyopadhyay R. Abera A. (2006a). Role of insects in the transmission of banana bacterial wilt. *Afr. Crop Sci. J.* 14 105–110.

[B190] Tinzaara W. Gold C. S. Tushmereirwe W. Bandyopadhyay W. Eden-Green S. (2006b). “The possible roles of insects in the transmission of banana *Xanthomonas wilt*,” in *Programme and Abstract Book of the 4th International Bacterial Wilt Symposium, 17th-20th July 2006* eds Saddler G. Elphinstone J. Smith J. (York: The Lakeland Conference Centre, Central Science Laboratory) 60.

[B191] Tripathi L. (2005). “Transgenic technologies for developing bacterial disease resistance in plants,” in *Genetic Resources and Biotechnology* Vol. 3 eds Thangadurai D. Pullaiah T. Tripathi L. (New Delhi: Regency Publications) 200–220.

[B192] Tripathi L. Mwaka H. Tripathi J. N. Tushemereirwe W. K. (2010). Expression of sweet pepper Hrap gene in banana enhances resistance to *Xanthomonas campestris* pv. musacearum. *Mol. Plant Pathol.* 11 721–731. 10.1111/j.1364-3703.2010.00639.x 21029318 PMC6640263

[B193] Tripathi L. Odipio J. Tripathi J. N. Tusiime G. (2008). A rapid technique for screening banana cultivars for resistance to *Xanthomonas wilt*. *Eur. J. Plant Pathol.* 121 9–19. 10.1007/s10658-007-9235-4

[B194] Tripathi L. Tripathi J. N. (2009). Relative susceptibility of banana cultivars to *Xanthomonas campestris* pv. musacearum. *Afr. J. Biotechnol.* 8 5343–5350.

[B195] Tripathi L. Tripathi J. N. Kiggundu A. Korie S. Shotkoski F. Tushemereirwe W. K. (2014). Field trial of *Xanthomonas wilt* disease-resistant bananas in East Africa. *Nat. Biotechnol.* 32 868–870. 10.1038/nbt.3007 25203031

[B196] Tripathi L. Tripathi J. N. Tushmereirwe W. K. (2004). Strategies for resistance to bacterial wilt disease of bananas through genetic engineering. *Afr. J. Biotechnol.* 3 688–692.

[B197] Turyagyenda L. F. Blomme G. Ssekiwoko F. Karamura E. Mpiira S. Eden-Green S. (2008). Rehabilitation of banana farms destroyed by *Xanthomonas wilt* in Uganda. *J. Appl. Biosci.* 8 230–235.

[B198] Tushemereirwe W. Kangire A. Smith J. Ssekiwoko F. Nakyanzi M. Kataama D. (2003). An outbreak of bacterial wilt on banana in Uganda. *InfoMusa* 12 6–8.

[B199] Tushemereirwe W. Kangire A. Ssekiwoko F. Offord L. C. Crozier J. Boa E. (2004). First report of *Xanthomonas campestris* pv. musacearum on banana in Uganda. *Plant Pathol.* 53:802 10.1111/j.1365-3059.2004.01090.x

[B200] Van Alfen N. K. (1989). Reassessment of plant wilt toxins. *Annu. Rev. Phytopathol.* 27 533–550. 10.1146/annurev.py.27.090189.002533

[B201] van Elsas J. D. van Overbeek L. S. Bailey M. J. Schönfeld J. Smalla K. (2005). “Fate of *Ralstonia solanacearum* biovar 2 as affected by conditions and soil treatments in temperate climate zones,” in *Bacterial Wilt Disease and the Ralstonia solanacearum Species Complex* eds Allen C. Prior P. Hayward A. C. (St. Paul, MN: APS Press) 39–49.

[B202] Vaneechoutte M. Kämpfer P. De Baere T. Falsen E. Verschraegen G. (2004). Wautersia gen. nov., a novel genus accommodating the phylogenetic lineage including *Ralstonia eutropha* and related species, and proposal of *Ralstonia* [Pseudomonas] syzygii (Roberts et al. 1990) comb. nov. *Int. J. Syst. Evol. Microbiol.* 54 317–327. 10.1099/ijs.0.02754-0 15023939

[B203] Vauterin L. Hoste B. Kersters K. Swings J. (1995). Reclassification of *Xanthomonas*. *Int. J. Syst. Bacteriol.* 45 472–489. 10.1099/00207713-45-3-472

[B204] Vauterin L. Yang P. Hoste B. Pot B. Swings J. Kersters K. (1992). Taxonomy of xanthomonads from cereals and grasses based on SDS-PAGE of proteins, fatty-acid analysis and DNA hybridization. *J. Gen. Microbiol.* 138 1467–1477. 10.1099/00221287-138-7-1467

[B205] Villa J. E. Tsuchiya K. Horita M. Opina N. Hyakumachi M. (2005). Phylogenetic relationships of *Ralstonia solanacearum* species complex strains from Asia and other continents based on 16S rDNA, endoglucanase, and hrpB gene sequences. *J. Gen. Plant Pathol.* 71 39–46. 10.1007/s10327-004-0156-1

[B206] Wang J.-F. Hanson P. Barnes J. A. (1998). “Worldwide evaluation of an international set of resistance sources to bacterial wilt in tomato,” in *Bacterial Wilt Disease: Molecular and Ecological Aspects* eds Prior P. Allen C. Elphinstone J. (Berlin: Springer-Verlag) 269–275.

[B207] Wardlaw C. W. (1972). *Banana Diseases Including Plantain and Abaca.* Harlow: Longman 878.

[B208] Wasukira A. Tayebwa J. Thwaites R. Paszkiewicz K. Aritua V. Kubiriba J. (2012). Genome-wide sequencing reveals two major sub-lineages in the genetically monomorphic pathogen *Xanthomonas campestris* pathovar musacearum. *Genes* 3 361–377. 10.3390/genes3030361 24704974 PMC3902798

[B209] Welde-Michael G. Bobosha K. Addis T. Blomme G. Mekonnen S. Mengesha T. (2008). Mechanical transmission and survival of bacterial wilt on enset. *Afr. Crop Sci. J.* 16 97–102.

[B210] Were E. (2016). *Endophytic Bacteria Associated with Banana and Their Potential for Controlling Banana Xanthomonas wilt.* A thesis for the award of the degree of Masters in Molecular Biology and Biotechnology, Makerere University Kampala.

[B211] Wicker E. Lefeuvre P. de Cambiaire J. C. Lemaire C. Poussier S. Prior P. (2012). Contrasting recombination patterns and demographic histories of the plant pathogen *Ralstonia solanacearum* inferred from MLSA. *Int. Soc. Microb. Ecol. J.* 6 961–974. 10.1038/ismej.2011.160 PMC3329105 22094345

[B212] Yabuuchi E. Kosako Y. Hano I. Hotta H. Nishiuchi Y. (1995). Transfer of two Burkholderia and an Alcaligenes species to Ralstonia gen. *nov*.: proposal of Ralstonia pickettii (Ralston, Palleroni, and Douderoff 1973) comb. nov., Ralstonia solanacearum (Smith, 1896) comb. nov. and *Ralstonia eutropha* (Davis 1969) comb nov. *Microbiol. Immunol.* 39 897–904. 10.1111/j.1348-0421.1995.tb03275.x 8657018

[B213] Yabuuchi E. Kosako Y. Oyaizu H. Yano I. Hotta H. Hashimoto Y. (1992). Proposal of *Burkholderia gen*. nov. and transfer of seven species of the genus Pseudomonas homology group II to the new genus, with the type species *Burkholderia cepacia* (Palleroni and Holmes 1981) comb. nov. *Microbiol. Immunol.* 36 1251–1275. 10.1111/j.1348-0421.1992.tb02129.x 1283774

[B214] Yirgou D. Bradbury J. F. (1968). Bacterial wilt of enset (*Ensete ventricosum*) incited by *Xanthomonas musacearum* sp. n. *Phytopathology* 58 111–112.

[B215] Yirgou D. Bradbury J. F. (1974). A note on wilt of banana caused by the enset wilt organism *Xanthomonas musacearum*. *East Afr. Agric. For. J.* 40 111–114.

[B216] Young J. M. Dye D. W. Bradbury J. F. Panagopoulos C. G. Robbs C. F. (1978). A proposed nomenclature and classification for plant pathogenic bacteria. *N. Zeal. J. Agric. Res.* 21 153–177. 10.1080/00288233.1978.10427397

[B217] Zehr E. I. (1970). Isolations of *Pseudomonas solanacearum* from abaca and banana in the philippines. *Plant Dis. Rep.* 54 516–519.

[B218] Zulperi D. Sijam K. (2014). First report of *Ralstonia solanacearum* Race 2 biovar 1 causing Moko disease of banana in Malaysia. *Plant Dis.* 98:275 10.1094/PDIS-03-13-0321-PDN 30708756

